# Tuning the Photophysics of Two-Arm Bis[(dimethylamino)styryl]benzene Derivatives by Heterocyclic Substitution

**DOI:** 10.3390/molecules27248725

**Published:** 2022-12-09

**Authors:** Letizia Mencaroni, Alessio Cesaretti, Benedetta Carlotti, Martina Alebardi, Fausto Elisei, Ana Ratković, Irena Škorić, Anna Spalletti

**Affiliations:** 1 Department of Chemistry, Biology and Biotechnology and Center of Excellence on Innovative Nanostructured Materials (CEMIN), University of Perugia, Via Elce di Sotto n.8, 06123 Perugia, Italy; 2 Department of Organic Chemistry, Faculty of Chemical Engineering and Technology, University of Zagreb, HR-10 000 Zagreb, Croatia

**Keywords:** fluorescence, solvatochromism, DFT quantum mechanical calculations, nonlinear optical properties, ultrafast spectroscopy, intersystem crossing, singlet oxygen sensitization

## Abstract

The identification of novel molecular systems with high fluorescence and significant non-linear optical (NLO) properties is a hot topic in the continuous search for new emissive probes. Here, the photobehavior of three two-arm bis[(dimethylamino)styryl]benzene derivatives, where the central benzene was replaced by pyridine, furan, or thiophene, was studied by stationary and time-resolved spectroscopic techniques with ns and fs resolution. The three molecules under investigation all showed positive fluorosolvatochromism, due to intramolecular charge-transfer (ICT) dynamics from the electron-donor dimethylamino groups, and significant fluorescence quantum yields, because of the population of a planar and emissive ICT state stabilized by intramolecular hydrogen-bond-like interactions. The NLO properties (hyperpolarizability coefficient and TPA cross-section) were also measured. The obtained results allowed the role of the central heteroaromatic ring to be disclosed. In particular, the introduction of the thiophene ring guarantees high fluorescent quantum yields irrespective of the polarity of the medium, and the largest hyperpolarizability coefficient because of the increased conjugation. An important and structure-dependent involvement of the triplet state was also highlighted, with the intersystem crossing being competitive with fluorescence, especially in the thiophene derivative, where the triplet was found to significantly sensitize molecular oxygen even in polar environment, leading to possible applications in photodynamic therapy.

## 1. Introduction

In our long-term research project on the push-pull behavior of stilbenoid compounds bearing donor–acceptor (D/A) groups linked by a conjugated bridge (D-π-A systems) [1,2,3,4,5,6], we mainly studied their emission and non-linear optical (NLO) properties [7,8,9,10,11,12] for applications as fluorescent sensors in the fields of optoelectronics, medicine, and communications [13,14,15,16,17]; the symmetric compounds (D-π-A-π-D or A-π-D-π-A) were found to show enhanced NLO capabilities when compared with the analogous dipolar structures [18,19,20,21,22,23,24].

These push-pull systems show large charge displacement during the excitation and the presence of intramolecular charge transfer (ICT) in the excited state particularly favored in polar solvents.

Recently we have studied the photobehavior of distyrylbenzene analogs in which the central benzene was replaced by a heteroaromatic ring (furan, pyridine, or thiophene), and the lateral benzenes functionalized with strong electron-withdrawing nitro groups in the para position [25,26].

These systems proved to be particularly interesting because of their symmetrical structure and given that in previous papers [27,28,29], it had been highlighted how the presence of the central heteroaromatic ring (either π-rich or π-deficient) strongly favored the electron transfer process by acting as an auxiliary A–D unit [30,31,32,33,34,35,36]. On the other hand, the nitro groups at the edge of the molecules were expected to produce a desirable bathochromic shift of the absorption spectra, marked CT character of the excited states and significant triplet production due to the introduction of ^3^(n,π*) states able to generate strong spin-orbit coupling [37,38,39,40,41].

Effectively, the latter compounds were found to show appreciable NLO properties adequate for NLO applications together with high photosensitized singlet oxygen production suitable for photodynamic therapy (PDT) as a non-invasive cancer treatment [25,26]. Of particular interest was the photobehavior of thiophene and furan derivatives which, alongside significant triplet and singlet oxygen yields, showed photostability and a remarkable ability to emit fluorescence [26]. In fact, photostable materials with such properties can be used as fluorescent photosensitizers for simultaneous cancer diagnosis (bioimaging) and therapeutic action (PDT) [42,43,44,45].

**Scheme 1:**
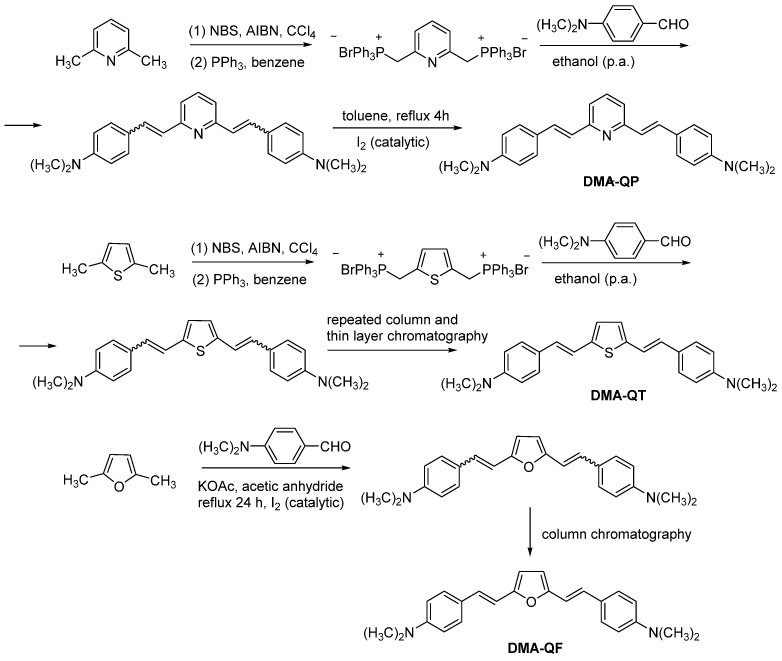
Synthetic scheme of the investigated compounds 2,6-bis[(*E*)-2-(4-aminophenyl)ethenyl]pyridine (DMA-QP), 2,5-bis[(*E*)-2-(4-aminophenyl)ethenyl]thiophene (DMA-QT) and 2,5-bis–[(*E*)-2-(4-aminophenyl)ethenyl]furan (DMA-QF).

In the present paper, the synthesis and photobehavior of *trans,trans* isomers of three bis[(dimethylamino)styryl]benzene derivatives (Scheme 1) are reported and compared to already-studied analogous nitro-distyrylbenzene derivatives with two nitro groups in the place of the two dimethylamino substituents.

A detailed experimental study using conventional steady-state and time-resolved (with ns and fs resolution) spectroscopic techniques coupled with theoretical calculations at the TD–DFT level was performed. The combined experimental and theoretical study allowed the competitive processes deactivating the excited electronic states and their mechanisms to be pointed out, highlighting the effect given by the central heteroaromatic ring (furan, pyridine, and thiophene). The spectral characterization of the three compounds in solvents of different polarity to derive their hyperpolarizability coefficient through a solvatochromic method is also reported, together with their two-photon absorption (TPA) cross-sections measured by the two-photon excited fluorescence technique.

Particular attention was paid to the comparison of the photobehavior of the compounds investigated of the present study, with the symmetric dinitro-derivative analogs and the asymmetric ones that carry both the dimethylamino group on one edge and the nitro group on the opposite side, with the intent to investigate how the replacement of strong electron-withdrawing nitro substituents with strong electron-donor dimethylamino ones can tune the photobehavior and NLO properties in these symmetrical systems.

## 2. Results

### 2.1. Solvent Effect on Spectral and Fluorescence Properties

**Figure 1:**
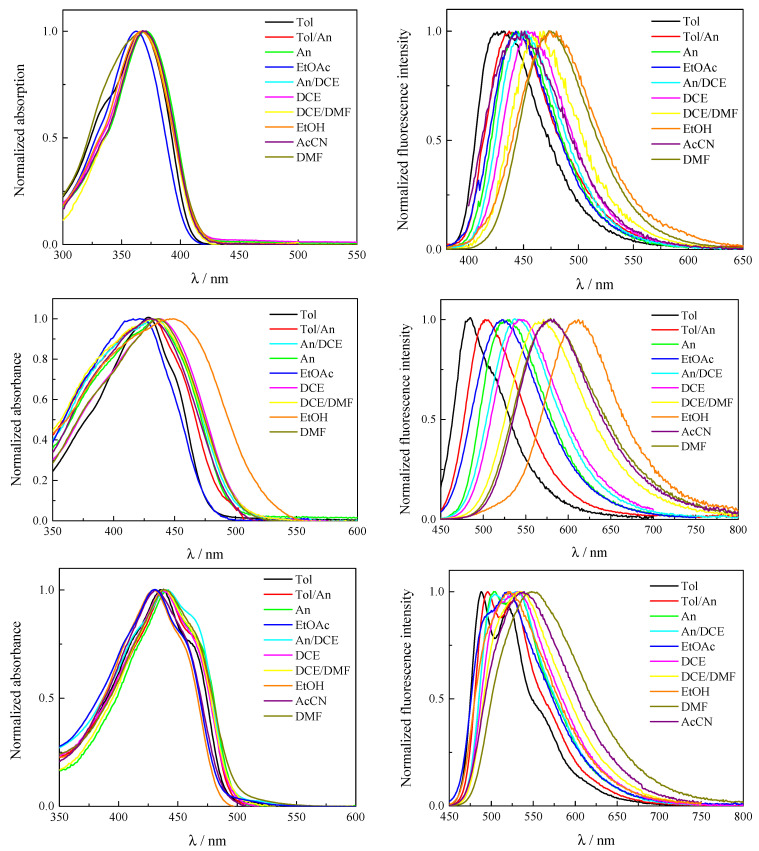
Absorption and emission spectra of compounds DMA-QP (**top**), DMA-QF (**middle**), and DMA-QT (**bottom**) in solvents of different polarity and polarizability. (Tol—toluene; An—anisole; EtOAc—ethyl acetate; DCE—1,2-dichloroethane; DMF—dimethylformamide; EtOH—ethanol; AcCN—acetonitrile).

The spectral characterization of the newly synthetized dimethylamino derivatives in many solvents of different polarity and polarizability is shown in Figure 1, while the molar absorption coefficients measured in Tol are reported in Table S1 and are all of the order of 30,000 M^−1^ cm^−1^. The absorption spectrum shifted to the red at about 60 to 70 nm when replacing the π-deficient pyridine unit with the π-rich furan and thiophene rings, respectively, due to the increased degree of conjugation [25,28]. DMA-QP showed an absorption maximum at around 370 nm, with a symmetric bell-shaped spectral profile; while a more structured shape was found in the cases of DMA-QF and DMA-QT, for which a bathochromic shoulder was also observed. The absorption spectra of the investigated compounds were barely affected by the solvent polarity, whereas the polarizability was responsible for the small blue-shift of the spectrum when dealing with low refractive index media, with the most hypsochromic absorption being recorded in EtOAc.

**Figure 2:**
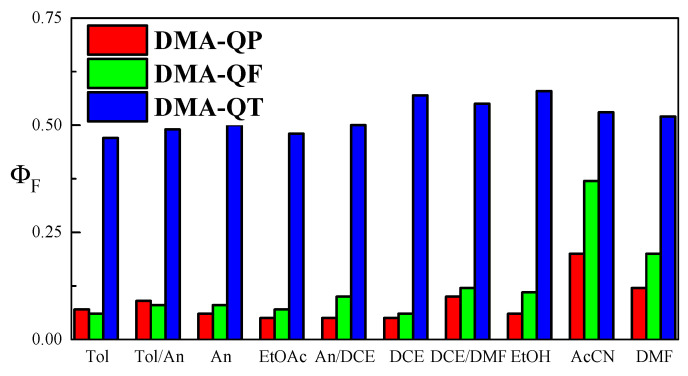
Fluorescence quantum yields of DMA derivatives in solvents of different polarity.

Contrarily, a marked fluorosolvatochromism emerged from the fluorimetric investigation, suggesting a higher dipolar character of the excited state when going towards more polar media [46]. The different nature of the emissive excited state, acquiring an intramolecular charge transfer (ICT) character, is also supported by the clear change of the emission profile, which features a net vibronic structure in Tol, particularly for DMA-QF and DMA-QT, and turning into a bell-like fluorescence spectrum in more polar environments. The fluorosolvatochromic behavior was mainly evidenced in the case of the furan derivative, with the emission maximum shifting to the red at about 130 nm moving from Tol to EtOH, as compared to 50 and ca. 30 nm found for DMA-QP and DMA-QT, respectively (see Table 1 below). Simultaneously, an increase of the Stokes shift and full width at half maximum (FWHM) upon increasing the solvent polarity was underscored, accompanied for DMA-QP and DMA-QF by a not negligible fluorescence quantum yield (Φ_F_ = 0.07 and 0.06 in Tol, respectively), which increased in polar media (Φ_F_ = 0.20 and 0.37 in AcCN, respectively, see Figure 2 below and Table S1). The only exception to this Φ_F_ trend was observed in the protic EtOH (Φ_F_ = 0.06 for DMA-QP and 0.11 for DMA-QF), for which the most bathochromically shifted emission was also found. In fact, the fluorescence quantum yield was halved compared to its value in DMF and was more than three times smaller than in AcCN for both compounds. This result could be linked to the presence of intramolecular hydrogen-like bonds between the central heteroatom and the ethylene hydrogens, which stabilize a fluorescent planar compressed form (see below, Section 2.2). In a protic solvent, intermolecular H-bonds loosen the intramolecular interactions, favoring less compressed and more distorted geometries, in which non-radiative deactivation pathways predominate [47,48]. The presence of thermal equilibria between many slightly different conformations in fluid media was also in line with the much broader absorption spectrum recorded for DMA-QF in EtOH, where each conformation contributed to the absorption with its distinct absorption profile (7400 cm^−1^ for the FWHM vs. 5900 cm^−1^ in DMF). The highest fluorescence quantum yields (Φ_F_ ~ 0.5) were measured in the case of DMA-QT, likely because of the more conjugated and planar structure conferred by the central less-aromatic thienyl ring. The emission capability of the thiophene-substrate, unlike the previous compounds, remained constant, being insensitive either to solvent polarity, polarizability, or even proticity, in line with the reduced tendency of the sulfur atom, as compared to nitrogen and oxygen, to form H-type bonds with solvent molecules. The weaker fluorescence of the first two systems in the series suggests that non-radiative pathways play a crucial role in their excited-state photodynamics (see below).

### 2.2. Quantum Mechanical Calculations

**Figure 3:**
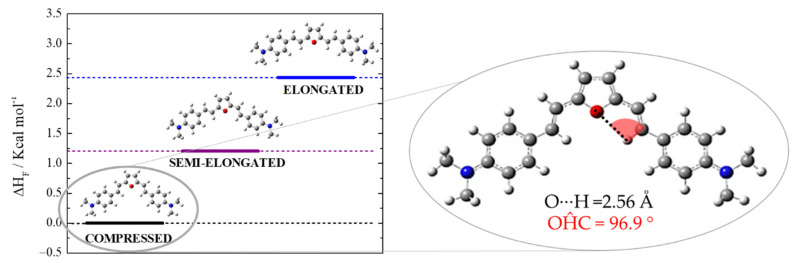
Relative energy of the ground-state conformers of DMA-QF in Tol (CPCM) calculated by the B3LYP/6–31 + G(d) model.

Notoriously, these double-arm molecules could exist in fluid solution at room temperature as dynamic equilibria among conformers originating by rotation around the quasi-single bonds between the heteroaromatic ring and the ethenyl bridge. DFT calculations aided in carrying out a complete theoretical study on the conformational equilibrium of the three DMA derivatives, in order to distinguish between the most abundant conformers likely present in solution—either the compressed form (dipolar-like), the semi-elongated or the elongated species (quadrupolar-like). From the optimizations of the ground- and excited-state geometries, the compressed form was found to largely prevail (see Figure 3 and Table S3) [49], and was also experimentally attested by the lack of wavelength effect on emission and fluorescence excitation spectra (see Figure S1 as a representative example), being stabilized by the intramolecular H-like bonds between the hydrogens of the adjacent ethenyl bridges and the central electronegative heteroatom (Figure 3). As a matter of fact, the intramolecular N⋯H/O⋯H distances of 2.54 and 2.56 Å, respectively, and the NĤC–OĤC angles (97.8° and 96.9°, respectively) demonstrate the H-bond type nature of this interaction and, thus, the prevalence of the bent structures (Table S4). For this reason, the pyridine and furan derivatives will be considered from now on to be dipolar-like push-pull systems with the dipole moment oriented along the y-axis. Adversely, the larger S—H distance (2.83 Å) and wider CĤS angle (102.9°), jointly with the more extended optimized structure, led to the interpretation of DMA-QT as a quadrupolar molecule (Table S4). In addition, the predicted excited-state dipole moments (Table S5), calculated through the Mulliken atomic charges and increasing upon excitation, showed the largest difference (Δμ_CT_) for the pyridine derivative and the lowest values for the ground and excited state in the case of the thiophene-substituted compound. In contrast, the Q_XX_ component of the quadrupole moment was found to slightly decrease upon excitation in the case of DMA-QP and DMA-QF, while it halved in the case of DMA-QT (Table S5). TD–DFT calculations (see Tables S6–S17) managed to successfully reproduce the experimental absorption spectra, whose main transition (S_0_ → S_1_) is significantly described by the π-π* HOMO-LUMO configuration (>70% for DMA-QP and >80% for DMA-QF and DMA-QT). The corresponding molecular orbitals (see Tables S7, S11, S14 and S16) showed a higher degree of ICT character for the relaxed excited state, which corroborates the occurrence, during the excited-state lifetime, of some charge displacement involving the central heterocycle and the ethenyl groups. This charge movement was more distinct in the case of the pyridine derivative, being a π-poor heterocycle in comparison to the furan and thiophene units. In addition, in the electron-density difference maps (Figures S2–S5), a consistent weakening of the double bonds is observed in the compressed DMA-QP when going from the S_0_ to S_1_ state, which likely favors trans-cis photoisomerization in the singlet manifold. As for DMA-QF, electron density depletion on the furan unit, with a consequent decrease in its aromaticity, is instead observed.

### 2.3. NLO Properties

**Figure 4:**
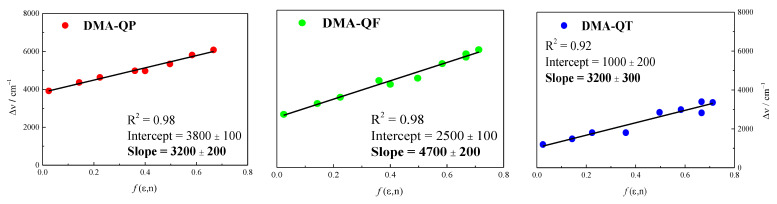
Linear plots of the Stokes shift vs. solvent properties (dielectric constant, ε, and refractive index, *n*) of the investigated compounds (DMA-QP (**left**), DMA-QF (**middle**), DMA-QT (**right**)).

The solvatochromic behavior showed by the three compounds under investigation was exploited in order to estimate second-order non-linear optical properties appealing for optoelectronic and photonics applications, quantified through the hyperpolarizability coefficients. The solvatochromic method has been successfully applied for push-pull compounds, proving to be a valid alternative and simplified way to explore those NLO properties without relying on more sophisticated techniques such as EFISH (electron-field induced second-harmonic) and HRS (hyper-Rayleigh scattering) [12,50,51]. From the slope given by the linear plot of the Stokes shift vs. the solvent properties (Figure 4), namely the dielectric constant and refractive index, derived by Equation (1), the experimental dipole-moment difference along the charge-transfer direction, Δμ_CT_, between the first excited singlet state and the ground state was determined. The Stokes shift recorded in EtOH was excluded from the plot for both DMA-QP and DMA-QF because of the intramolecular H-type bonds with the solvent affecting spectral positions beside solvatochromism. The Δμ_CT_ values match the fluorosolvatochromic behavior of the DMA derivatives; the largest difference was found for the furan-derivative, followed by the pyridine- and thiophene-substituted compounds. This information was then manipulated using Oudar’s formula (Equation (2)) for the pyridine and furan derivatives and in the modified equation (Equation (3)) for the quadrupolar DMA-QT compound, to give the dynamic hyperpolarizability coefficients (β_CT_) in Tol (see Table 2). The sizeable β_CT_ values (248, 924, and 1030 esu^−1^ cm^5^ for DMA-QP, DMA-QF, and DMA-QT, respectively) were found to be comparable to other NLO materials and their trend reproduces the increased conjugation when replacing the central pyridine with the furan and, particularly, with the thienyl unit, as found for other push-pull molecules [51].

**Figure 5:**
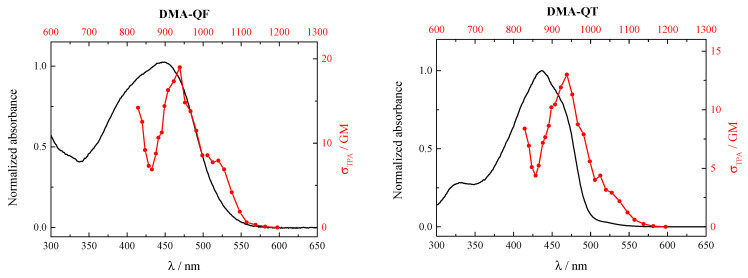
Comparison between TPE (two-photon excitation, red) and OPE (one-photon excitation, black) spectra of compounds DMA-QF (**left**) and DMA-QT (**right**) in DMF obtained by TPEF technique.

The considerable emission capability of the three molecules under study enabled the application of the two-photon excited fluorescence (TPEF) technique to probe the third order NLO properties and, thus, measure the two-photon cross-sections (σ_TPA_). The two-photon excitation (TPE) spectra (Figure 5) were obtained by exciting the sample (DMA-QF and DMA-QT in DMF) with two simultaneous photons of halved frequency, tuning the excitation radiation in the 830–1200 nm range, and acquiring the fluorescence excitation spectrum to be compared with the one-photon excitation (OPE) spectral profile. Given its hypsochromic absorption spectrum and the accessible spectral window in the employed experimental setup, the same experiment could not be carried out in the case of DMA-QP. The obtained σ_TPA_ values were on the order of tens of GM. The TPE spectrum, in red, was found to differ from the black OPE profile in both investigated compounds (see Figure 5). However, this result is not surprising as distinct selection rules govern OPA and TPA electronic transitions, modifying transition probabilities, which are also sensitive to molecular symmetry. For this reason, the blue edge of the TPE spectra may envisage the onset of one-photon partially forbidden but two-photon allowed S_0_ → S_2_ transitions predicted at _OPA_ = 317 and 323 nm (see Tables S10 and S15). The mismatch between the TPE and OPE maxima may also imply that different conformations might be preferentially excited with the TPEF technique, resulting in a slightly red-shifted fluorescence–excitation profile. As a matter of fact, the TPA process can be influenced by conformation changes as it correlates to the direction and magnitude of the transition dipole moment, which may differ when dealing with distinct conformations [52,53].

### 2.4. Excited-State Dynamics

**Figure 6:**
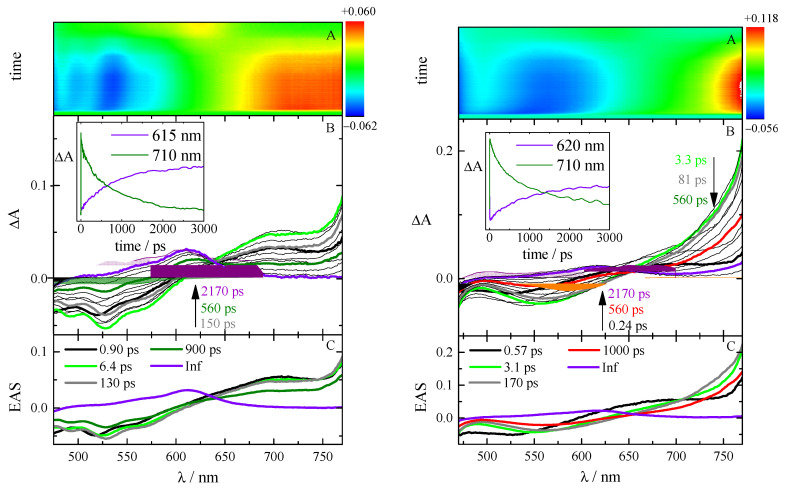
Femtosecond transient absorption measurements of compound DMA-QT in Tol (**left**) and DMF (**right**) obtained by pump-probe experiment (λ_exc_ = 400 nm). Panel (**A**), experimental 3D matrix reporting color-coded ΔA as a function of wavelength and time; Panel (**B**), representative spectra at different delay times and representative kinetics (inset) at different wavelengths, together with steady-state absorption (gray-shaded area), emission (green-shaded area or orange-shaded area), and triplet TA spectra (purple-shaded area as obtained by ns laser flash photolysis shown below); Panel (**C**), EAS (excited associated spectra) obtained by global analysis.

Ultrafast transient absorption (TA) spectroscopy was applied to shed light on the excited-state deactivation dynamics of the three investigated molecules in solvents of different polarities and to draw an overall picture of the competitive decay channels involved. The experimental results were subsequently rationalized through global analysis in order to identify all the transient species, together with their lifetimes and evolution-associated spectra (EAS). The relative results for the three molecules under study are compiled in Table 3 and Table S19. Generally, the time-resolved transient spectra of the three DMA derivatives (Figure 6, Figures S6 and S7) featured two kinds of footprints in the explored spectral window (450–780 nm); first, a broad positive excited-state absorption (ESA) band, referring to the S_1_→S_n_ transitions; second, a negative signal perfectly matching the steady-state emission spectrum, thus attributed to the stimulated emission (SE). The fs-TA spectrum of DMA-QT in Tol is reported as a case study in Figure 6. Here, the global analysis pointed out five transients (Panel C); the first four spectral profiles share many common features, a broad peak at ca. 700 nm, formed in <1 ps, and a structured stimulated emission signal at ca. 575 nm. A closer look reveals that the emission minimum is slightly red-shifted when going from the black transient to the gray profile. This evolution is the well-known solvation (inertial, black, and diffusional, light green) dynamics [54,55], followed by further stabilization due to the structural relaxation (SR) of the molecule in the new excited configuration. The fourth transient, resembling the previous ones, is the more stabilized, emissive S_1_ state, overlapping the steady-state fluorescence (green-shaded area in Figure 6, left). The violet transient, centered at 610 nm and appearing at later delays, was instead ascribed to the long-lived T_1_ state. This assignment is justified by the following evidence: the transient overlaps the spectral shape resulting from nanosecond transient absorption measurement (purple-shaded area in Figure 6, left, see below); from the representative kinetics (Figure 6, inset of Panel B), the rise time of this transient (kinetic at 615 nm) is concomitant with the S_1_ decay (kinetic at 710 nm); and no trace of decay was found, meaning that its lifetime far exceeds the experimental time window of 3.2 ns. The rise–decay kinetic can be therefore interpreted as the occurrence of an ISC process. Fluorescence up-conversion (FUC) experiments corroborate the results given by the complementary transient absorption. As a matter of fact, all the aforementioned transients with almost equal lifetimes were identified by the FUC measurements, except for the absence of the dark triplet state (Figures S8–S10). The global analysis of these experimental data then showed that DMA-QT displayed the longest S_1_ lifetimes (τ_S1_ = 840 ps in Tol), around one order of magnitude longer than those obtained for the pyridine and furan-analogs (τ_S1_ = 89 and 93 ps, respectively), in line with the highest fluorescence quantum yield in the DMA-derivative series.

**Figure 7:**
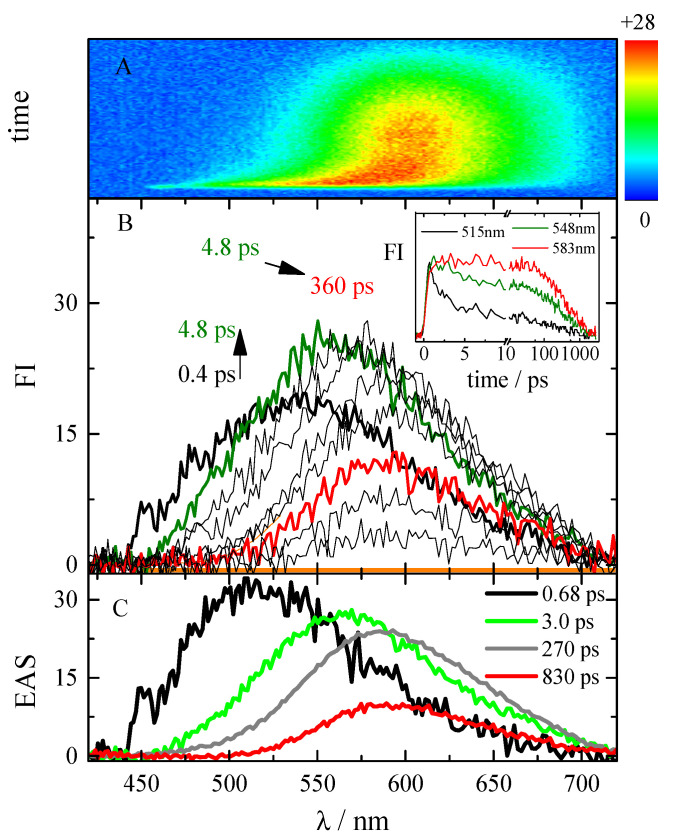
Femtosecond fluorescence up-conversion measurements (λ_exc_ = 400 nm) of compound DMA-QF in DMF. Panel (**A**): experimental 3D matrix reporting color-coded fluorescence intensity (FI) as a function of wavelength and time; Panel (**B**): representative emission spectra at different delay times and representative kinetics (inset) at different wavelengths together with steady-state emission (orange-shaded area); Panel (**C**): EAS (excited associated spectra) obtained by global analysis.

When it comes to a more polar environment, the photobehavior deduced from the TA experiment is described by the same transients listed above, which are the two solvation components and the structural relaxation (hundreds of picoseconds), with the only exception of a new species, reported in red (see Figure 6, right, and Figures S6 and S7). This species not only presented a blue-shifted ESA band relative to the Franck–Condon singlet excited state, namely the locally excited (LE) state, but also a red-shifted SE signal, resembling the steady-state fluorescence spectrum in polar media (orange-shaded area in the relative figures). Its emissive nature was also confirmed by the fluorescence up-conversion measurements (see Figure 7 for DMA-QF in DMF as a reference example and Figures S8 and S10 for the remaining compounds). The marked reddening (ca. 100 nm) of the time-resolved emission EAS spectra (Figure 7, Panel C) over time in polar DMF, showing the stabilization of the emissive state, draws attention to the enhanced ICT character of relaxed S_1_. The change in the singlet excited-state nature, S_1,LE_ → S_1,ICT_, for all the investigated compounds of the series occurred during the first ultrafast transient species (<2 ps), that is during the inertial solvation. Consequently, the newly formed and more polar singlet state was progressively stabilized until the relaxed structure was reached, resulting in the low-lying fluorescence emission at longer wavelengths. Analogous to what was observed in the steady-state fluorimetric measurements, the largest red-shift of the S_1,ICT_ was found for DMA-QF, while it was only hinted at in the case of the other two compounds. As observed for other symmetrical push-pull compounds [26,41,56], the singlet lifetimes in the cases of DMA-QP and DMA-QF increased one order of magnitude when going from a non-polar solvent such as Tol towards a polar medium (τ_S1_ = 970 and 830 ps in DMF, respectively), implying the involvement of a planar ICT state and matching the parallel increase of the Φ_F_. This result goes along with the decrease of triplet production in a polar solvent (see below), where the stabilization of the S_1,ICT_ is detrimental to the population of the triplet state. The lifetimes of the emissive excited states obtained from the FUC measurements were employed to determine the fluorescence kinetic constants (k_F_) in both non-polar and polar media (Table S2). The k_F_ values were found to be ~10^8^ s^−1^ in both Tol, as also inferred by the large oscillator strengths calculated for the S_1_→S_0_ transition (Tables S6–S18) and DMF, highlighting a fully-allowed process even when the excited state turns into an ICT state. However, a small drop in k_F_ was observed for both DMA-QP and DMA-QF due to the gain in internal conversion originating from the stabilized ICT state.

**Figure 8:**
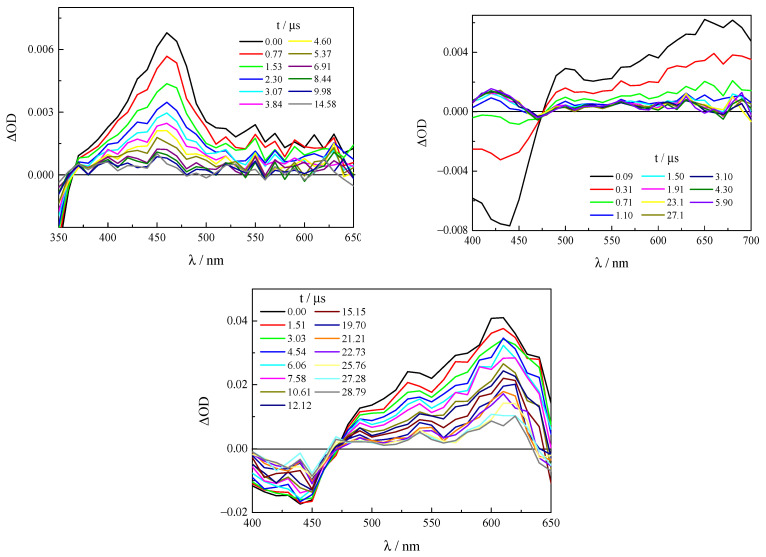
Nanosecond transient absorption spectra of DMA-QP (**top left**), DMA-QF (**top right**), and DMA-QT (**bottom**) in de-aerated Tol solutions obtained by laser flash photolysis (λ_exc_ = 355 nm).

Nanosecond laser-flash photolysis measurements in aerated and de-aerated Tol (Figure 8 and Figure S12) and DMF (Figure S11) solutions provide details on the triplet excited-state properties of the DMA derivatives under investigation, particularly the transient absorption spectra, triplet lifetimes, and quantum yields. For all the investigated compounds, the triplet formation was relatively fast (k_T_ ~ 10^8^–10^9^ s^−1^, Table 4). TD–DFT calculations indeed revealed for DMA-QP an S_0_ → T_3_ transition described by a HOMO-2 → LUMO + 1 configuration almost isoenergetic with the S_0_ → S_1_ transition but showing lower charge-transfer character (Tables S6 and S7, and Figure S2). The presence of this triplet excited state in close proximity to S_1_ may favor ISC and thus be the reason for the significant triplet kinetic constant (k_T_ = 1.2 × 10^9^ s^−1^). As for the furan and thiophene derivatives, for which the abovementioned transition was also predicted but at much higher energies (Tables S10 and S15), the noticeable values obtained for the k_T_ process may be explained by the presence of the heavier central atoms. The ns-TA spectrum recorded in Tol is constituted by two distinct signals (Figure 8): a positive ESA band, peaked at 460 nm for DMA-QP, 650 nm for DMA-QF, and 610 nm for DMA-QT in N_2_-purged solution, which is barely affected by solvent polarity (with the only exception of a 10 nm-blue shift in the case of the pyridine-derivative in DMF); and a hypsochromic, negative signal (<350 nm for DMA-QP, centered at 430 nm for DMA-QF and 440 nm for DMA-QT) overlapping the steady-state absorption. The latter was hence assigned to ground-state bleaching (GSB), while the positive ESA band was interpreted as a T_1_ → T_n_ signal given that its formation took place during the laser pulse, it followed a first-order decay kinetic, and molecular oxygen quenched its lifetime. This assignment was also validated by sensitization experiments from high-energy triplet donors (DTK) or energy transfer to low-energy triplet acceptors (D2TO). An additional positive signal was detected at later delays in the case of DMA-QF at ca. λ_T_ = 420 nm in Tol and ca. λ_T_ = 470 nm in DMF (Figure 8 and Figure S11). Considering the rise–decay kinetics, with the rise time matching the very short decay time (τ_T,N2_ = 0.41 μs in Tol, see Table 4) of the ESA at 650 nm, and given its blue-shifted position relative to the GSB, the delayed signal might be tentatively assigned to the cis-trans isomer of the furan derivative, thus formed by rotation of one double bond from a highly reactive triplet state. DMA-QF faced the most marked solvent effect; the triplet production was 0.41 in Tol, but then dropped below the detection limit in DMF. The ultrashort τ_T_ combined with a very low ∆OD limited the quantitative characterization of the triplet state in this solvent. Similarly, the modest triplet production yield measured for DMA-QP in a non-polar solvent (Φ_T_ = 0.11 in Tol) and the short triplet lifetime, together with low fluorescence efficiency, suggest a dominant role of reactivity even in the excited-state deactivation dynamics of the pyridine substrate. In fact, when the non-radiative kinetic ($k_{nr}=\frac{1-\Phi_{F}-\Phi_{T}}{\tau_{F}}$) constants are considered (Table S2), high values of the order of ~10^9^–10^10^ s^−1^ are found for DMA-QF and DMA-QP in both non-polar and polar media, pointing to a major contribution of non-radiative decay channels (i.e., isomerization and internal conversion) for their excited singlet states.

On the contrary, the quadrupolar-like thiophene analog showed longer triplet lifetimes (tens of microseconds) with high triplet quantum yields both in Tol (Φ_T_ = 0.80) and in DMF (Φ_T_ = 0.43). In the latter solvent, the sum of fluorescence and intersystem-crossing quantum yields add up to almost the entirety, pointing to the absence of other deactivation channels for DMA-QT in a polar medium. When it comes to Tol, this value exceeded 100% (Φ_F_ + Φ_T_ = 1.3), thus entailing some peculiar phenomenon that was worth analyzing. The effect of concentration on the emission spectra were thus evaluated up to the conditions reached in the flash photolysis experiments, which require about one order of magnitude more highly concentrated solutions. In fact, self-absorption phenomena were revealed (Figure S13) and the fluorescence quantum yield was found to decrease from 0.51 to 0.27 with concentration. This implies that in a concentrated solution the compound is caught in some sort of radiative trapping, where the absorbed energy is transferred radiatively to a nearby molecule contributing to the quantitative population of the triplet state. In addition, the DMA-QT excited triplet state proved capable of efficiently sensitizing singlet molecular oxygen (Φ_∆_ ~70%, Figure S14), making this compound alluring for PDT application.

## 3. Discussion

The study of the trans,trans isomers of the three bis[(dimethylamino)styryl]benzene derivatives under investigation proved very informative about the role of the central heteroatoms in tuning the spectral, photophysical, and NLO properties of this molecular series. In fact, the π-rich furan and thiophene rings endow the system with an increased degree of conjugation, shifting the absorption spectra towards longer wavelengths as opposed to the absorption of DMA-QP, which instead features a π-deficient pyridine ring. Conjugation also plays a leading role in determining the entity of the hyperpolarizability coefficients, which were found to be large and comparable when dealing with DMA-QF and DMA-QT, due to the great electron-transport ability of furan and thiophene [32], and about four times lower in the case of the pyridine derivative. On the other hand, this behavior revealed a modest contribution offered by charge transfer to second-order NLO properties; fluorosolvatochromism and excited-state dynamics indeed revealed a higher ICT degree (higher Δμ_CT_) for the relaxed excited state of the DMA-QF derivative, but this did not increase its β value as compared to that of DMA-QT (β = 920 and 1030 10^−30^ esu^−1^ cm^5^, while Δμ_CT_ = 27.6 and 21.1 D for DMA-QF and DMA-QT, respectively). Interestingly, the close dependence of the β value on conjugation has also been corroborated by previous results on push-pull cationic chromophores [34,51], as well as on symmetric and asymmetric styrylbenzene derivatives [25,57], analogs of those reported in this work, whose hyperpolarizabilities were always found to be on the order of 1000 × 10^−30^ esu^−1^ cm^5^ either when symmetrically substituted with two nitro groups or when bearing nitro and dimethylamino substituents at the two opposite sides of the molecule, without altering the conjugation of the central structure. These values were instead halved in the case of an asymmetric substitution with a nitro and a methoxy group (460 and 420 esu^−1^ cm^5^ for the furan and thiophene derivatives, respectively), which indeed boosts the ICT degree (Δμ_CT_ = 34.6 and 31 D for the furan and thiophene derivatives, respectively) but reduces conjugation, thus affecting the β value [57].

Conversely, the ICT character is fundamental for enhancing third order NLO properties, such as TPA abilities, with DMA-QF exhibiting higher TPA cross-sections relative to DMA-QT. The σ_TPA_ were, however, modest and the TPE spectra did not overlap the OPE profiles. This finding could be explained by taking into account the conformational equilibria between compressed and elongated forms. On the one hand, the compressed and bent configuration is favored because of intramolecular hydrogen-like bonds; on the other hand, the elongated form is expected to show a higher TPA cross-section because of the larger transition dipole moment associated with a straight configuration, as already reported for other systems [53], thus contributing to the redshift of the TPE spectrum. In fact, the existence of other configurations also becomes apparent when DMA-QF is studied in a protic solvent like EtOH, where intermolecular hydrogen bonds can be introduced between the heteroatom and solvent molecules, resulting in the notable broadening of the absorption spectrum.

The presence of ICT dynamics for the three molecules was evidenced by the important fluorosolvatochromism, especially marked for DMA-QF, and the significant spectral evolution probed by fs-resolved ultrafast spectroscopy. However, the lack of excited-state lifetime quenching with solvent polarity, which was instead found to increase for DMA-QP and DMA-QF when passing from a scarcely to a highly polar medium because of the competition with intersystem crossing, points to a planar ICT state which remains significantly fluorescent. The emissive capability in a polar environment differed radically from what has been previously observed for asymmetric styrylbenzene analogs, whose highly dipolar push-pull A-π-D structure leads to the formation of an ICT state that becomes non-emissive upon increasing polarity [29]. As for the DMA-QP derivative, its non-negligible emission efficiencies even in a highly polar water–AcCN mixture have already been exploited to unravel an interesting acido(fluoro)chromic behavior, upon a first protonation on the central pyridine followed by a second protonation involving the lateral dimethylamino substituent [58]. As a rule, the fluorescence quantum yields of the three bis[(dimethylamino)styryl]benzene derivatives in polar media were always higher than those of the bis(nitrostyryl)benzene analogs (Φ_F_ = 0.20, 0.37, and 0.53 for DMA-QP, DMA-QF, and DMA-QT, respectively, vs. Φ_F_ = 0.004, 0.10, and 0.21 for the corresponding dinitro compounds) [26], making the former better emissive probes with a view to possible fluorescence imaging applications in a biological environment. In this sense, the triplet properties also awaken deep interest in the DMA-QT molecule. While DMA-QP and DMA-QF experienced a drop for the triplet quantum yield in polar DMF, where the stabilization of the ICT state put the triplet out of reach, the intersystem crossing in DMA-QT remained very efficient with increasing polarity, with Φ_T_ = 0.43 and very significant production of singlet oxygen by sensitization from the triplet. These properties make the dimethylamino-thiophene derivative even more attractive than its nitro-substituted counterpart, where both fluorescence and triplet production were found to reduce with polarity [26].

DMA-QT, therefore, features a very interesting photobehavior in a polar environment, where its excited state deactivates entirely by fluorescence and intersystem crossing (followed by molecular oxygen sensitization) in an almost 50/50 ratio, making the molecule promising for applications as both an imaging probe and a PDT agent.

## 4. Materials and Methods

### 4.1. Synthesis

The synthesis procedure of the DMA-QP compound has been already described in a previous paper [58], while those regarding the furan and thiophene derivatives are reported below.

**DMA-QF (4,4′-((1*E*,1′*E*)-furan-2,5-diylbis(ethene-2,1-diyl))bis(*N*,*N*-dimethylaniline).** To a stirred solution of the 2,5-dimethylfuran in acetic anhydride (3 eq), *p*-dimethylaminobenzaldehyde (2 eq, 0.45 mmol), KOAc (1 eq), and acetic anhydride (3 eq) were added. In addition, a catalytic amount of I_2_ was added and the reaction mixture was left on reflux for 24 h. The reaction mixture was poured into the ice and saturated with aqueous NaOH. The precipitate was formed, filtered, and washed with water. After removal of the solvent, the residue was worked up with water and toluene and dried over MgSO_4_. The crude reaction product was chromatographed and the pure *E,E-*isomer of DMA-furan was obtained in the last fractions using petroleum ether–diethylether (5%) mixture as eluent.

**DMA-QF**: yellow solid; isolated yield 21% (32 mg); *R_f_* = 0.35 (petroleum ether–diethylether (5%)); m.p. 119–122 °C; ^1^H NMR (CDCl_3_, 600 MHz) *δ*/ppm of 7.37 (d, 4H, *J* = 8.7 Hz), 6.93 (d, 2H, *J* = 16.5 Hz), 6.72 (d, 4H, *J* = 8.7 Hz), 6.68 (d, 2H, *J* = 16.5 Hz), 6.09 (s, 2H), 2.97 (s, 12H); ^13^C NMR (CDCl_3_, 150 MHz) *δ*/ppm of 152.1 (s), 150.8 (s), 149.4 (s), 128.8 (2d), 125.4 (2d), 112.1 (d), 107.5 (d), 107.1 (d), 39.9 (2q); HRMS for C_24_H_26_N_2_O: M^+^_calcd_ 358.2045; M^+^_found_ 358.2049.

**DMA-QT (4,4′-((1*E*,1′*E*)-thiophene-2,5-diylbis(ethene-2,1-diyl))bis(*N*,*N*-dimethylaniline)).** To a stirred solution of 2,5-dimethylthiophene in tetrachloromethane, *N*-bromosuccinimide (0.6 eq) and azobisisobutyronitrile (0.1 eq) were added. The reaction mixture was heated on reflux and irradiated with a halogen lamp (75 W) overnight. After cooling down to RT, the reaction mixture was filtrated to remove succinimide and evaporated. After the removal of the solvent, an extraction with dichloromethane and water was carried out. The extract was dried and concentrated. The crude product was dissolved in benzene and triphenylphosphine (PPh_3_) in benzene was added. After stirring overnight at RT the precipitate was filtered off and used in the next step after drying. To a stirred solution of obtained phosphonium salt (0.45 mmol) and *p*-dimethylaminobenzaldehyde (0.45 mmol) in p.a. ethanol, sodium ethoxide was dropwise added (10.3 mg, 0.45 mmol of Na dissolved in 5 mL p.a. ethanol). Stirring continued for 4 h at RT. After removal of the solvent, the residue was worked up with water and toluene and dried over MgSO_4_. The crude reaction product was chromatographed and the pure *E,E*-isomer of DMA-thiophene was obtained in the last fractions (after minor quantities of *Z,Z*-isomer and *Z,E*-isomer as the major one) by repeated column and thin-layer chromatography using petroleum ether–diethylether mixture as eluent (5%).

**DMA-QT**: yellow solid; isolated yield 14% (25 mg); *R_f_* = 0.30 (petroleum ether–diethylether (5%)); m.p. 128–130 °C; ^1^H NMR (CDCl_3_, 600 MHz) *δ*/ppm of 7.36 (d, 4H, *J* = 9.1 Hz), 6.99 (d, 2H, *J* = 16.3 Hz), 6.84 (s, 2H), 6.82 (d, 2H, *J* = 16.3 Hz), 6.70 (d, 4H, *J* = 9.1 Hz), 2.99 (s, 12H); ^13^C NMR (CDCl_3_, 150 MHz) *δ*/ppm of 146.8 (s), 141.8 (s), 137.9 (s), 127.4 (d), 127.2 (2d), 125.6 (2d), 124.8 (d), 112.5 (d), 40.4 (2q); HRMS for C_24_H_26_N_2_S: M^+^_calcd_ 374.1817; M^+^_found_ 374.1814.

### 4.2. Synthesis Characterization

The ^1^H and ^13^C NMR spectra were recorded on a spectrometer at 600 MHz (Figures S15–S18). All NMR spectra were measured in CDCl_3_ using tetramethylsilane as a reference. High-resolution mass spectra (HRMS) were obtained on a matrix-assisted laser desorption/ionization time-of-flight MALDI-TOF/TOF mass spectrometer (4800 Plus MALDI-TOF/TOF analyzer, Applied Biosystems Inc., Foster City, CA, USA) equipped with Nd:YAG laser operating at 355 nm with a firing rate of 200 Hz in the positive ion reflector mode, described in detail in Ref. [58]. A total of 1600 shots per spectrum were taken with a mass range of 100–1000 Da, a focus mass of 500 Da, and a delay time of 100 ns. Nicotinamide and azithromycin were used for external mass calibration in positive ion mode.

### 4.3. Materials

The structures of the molecules under investigation are presented in Scheme 1. Cyclohexane (CH, VWR Chemicals, Radnor, PA, USA)), toluene (Tol, ACS Reagent, Sigma-Aldrich, St. Louis, MO, USA), anisole (An, Sigma-Aldrich) ethyl acetate (EtOAc, AnalaR, BDH, Mumbai, India), 1,2-dichloroethane (DCE, ACS Reagent, Sigma-Aldrich), dimethylformamide (DMF, ACS Reagent, Sigma-Aldrich), ethanol (EtOH, VWR Chemicals) and acetonitrile (AcCN, VWR Chemicals) of spectroscopic grade were purchased for the spectral and photophysical characterizations.

### 4.4. Photophysical Measurements

Absorption spectra were recorded with a Cary 4E (Varian) spectrophotometer. Fluorescence emission and excitation spectra were detected using a FluoroMax-4P (HORIBA Scientific) spectrofluorimeter and analyzed by FluorEssence software with appropriate instrumental response correction files. The fluorescence quantum yields (Φ_F_, experimental error ± 10%) of dilute solutions (A at λ_exc_ < 0.1) were obtained by exciting each sample at the relative maximum absorption wavelength by employing 2-(1-naphthyl)-5-phenyl-1,3,4-oxadiazole (α-NPD), 9,10-diphenylanthracene and tetracene (Φ_F_ = 0.70 [59], 0.73 and 0.17 in air-equilibrated cyclohexane [60], respectively) as reference compounds.

Singlet oxygen quantum yields (Φ_Δ_) were measured in air-equilibrated Tol and DMF solutions using the DMA derivatives as sensitizers. The ^1^O_2_ phosphorescence spectra were detected using a spectrofluorimeter FS5 (Edinburgh Instrument) equipped with an InGaAs detector. Phenalenone (Φ_Δ_ = 0.99 and 1.00 in Tol and DMF, respectively) was employed as a reference compound for comparison purposes [61].

Even though approximated, the solvatochromic method managed to give a valid estimation of the second order hyperpolarizability coefficients of β_CT_ values measured by the more sophisticated EFISH (electric-field-induced harmonic generation) or HRS (hyper–Rayleigh scattering) techniques, with the advantages of simplicity and the application of conventional steady-state stationary spectroscopy only. As for the case of the EFISH method, the solvatochromic method provides the β_CT_ dominant contribution, which corresponds to the β_XXX_ component of the β tensor describing the CT transition and being referred to as the frequency of the exciting laser of EFISH allows for direct comparison and good agreement between the results of the two approaches.

This method is based on the results of the fluorosolvatochromic behavior. The dependence of the Stokes shift ($\Delta \tilde{\nu }$) from the solvent properties (dielectric constants, ε, and refractive index, *n*) expressed as $f\left(\varepsilon ,n\right)=\left(\frac{\varepsilon -1}{\varepsilon +2}-\frac{n^{2}-1}{n^{2}+2}\right)$ retains the information about the dipole-moment difference between the ICT excited state and the ground state (Δμ_CT_ = μ_E_ − μ_G_) according to Equation (1):

(1) $$\Delta \tilde{\nu }=\tilde{\nu_{abs}}-\tilde{\nu_{em}}=\left(\delta_{abs}-\delta_{em}\right)+\frac{2\Delta \mu_{CT}{}^{2}}{hca^{3}}\left(\frac{\varepsilon -1}{\varepsilon +2}-\frac{n^{2}-1}{n^{2}+2}\right)$$

where $\Delta \tilde{\nu }=\tilde{\nu_{abs}}-\tilde{\nu_{em\;}}$ is the Stokes shift (in cm^−1^), *a* is the cavity radius within Onsager’s model, taken as 60% of the calculated diameter along the CT direction resulting from the optimized geometry [62] in cm, *h* is the Planck constant and *c* is the speed of light in a vacuum.

In the case of DMA-QT, as also reported for other quadrupolar push-pull systems [12,25], another equation was applied that relates the stokes shift to the quadrupole moment difference (ΔQ = Q_E_ − Q_G_) as follows:

(2) $$\Delta \tilde{\nu }=\tilde{\nu_{abs}}-\tilde{\nu_{em\;}}=\left(\delta_{abs}-\delta_{em}\right)+\frac{2Q_{CT}{}^{2}}{hca^{5}}\left(\frac{\varepsilon -1}{\varepsilon +2}-\frac{n^{2}-1}{n^{2}+2}\right)$$

where Q = 2µ*d* and thus ΔQ_CT_ = 4d^2^(Δμ_CT_)^2^. In this case, the quadrupole is considered as two opposite dipoles, separated by a distance *d* between their barycenter, sharing the central heteroatomic ring.

The dynamic hyperpolarizability coefficient was then derived through Oudar’s formula [13]

(3) $$\beta_{CT}=\beta_{xxx}=\frac{3}{2h^{2}c^{2}}\times \frac{{\tilde{v}}_{eg}{}^{2}r_{eg}{}^{2}\Delta \mu_{CT}}{\left({\tilde{v}}_{eg}{}^{2}-{\tilde{v}}_{L}{}^{2}\right)-\left({\tilde{\nu }}_{eg}^{2}-4{\tilde{\nu }}_{L}^{2}\right)}$$

where $r_{eg}$ is the transition dipole moment related to the oscillator strength (*f*) obtained by the integrated absorption band as $f=4.32\times 10^{-9}\int \varepsilon \left(v\right)dv$ [63] or predicted by quantum mechanical calculations, ${\tilde{\nu }}_{eg}$ is the maximum frequency (in cm^−1^) of the bathochromic CT transition, and ${\tilde{\nu }}_{L}^{}$ is the frequency of the incident radiation, chosen as 1064 nm of Nd:YAG laser, to which the β values would be referred. The static, frequency-independent hyperpolarizability coefficient β_0_ has been calculated considering Equation (4) [64]:

(4) $$\beta_{0}=\frac{3}{2h^{2}c^{2}}\frac{r_{eg}{}^{2}\Delta \mu_{CT}}{{\tilde{\nu }}_{eg}^{2}}$$

The experimental setup for the femtosecond transient absorption and fluorescence up-conversion measurements have been widely described elsewhere [3,24,27]. Briefly, the 400-nm excitation pulses of about 60 fs were generated by an amplified Ti–Sapphire laser system (Spectra Physics, Mountain View, CA, USA). The transient absorption spectrometer (Helios, Ultrafast Systems, Sarasota, FL, USA) was characterized by 150 fs time and 1.5 nm spectral resolutions. Probe pulses were produced in the 450–750 nm range by passing a small fraction of the 800 nm excitation radiation through an optical delay line (time window of 3200 ps) and focusing it onto a Sapphire crystal (2 mm thick) to generate a white-light continuum. In the fluorescence up-conversion setup (Halcyone, Ultrafast System), the 400-nm pulse excites the sample while the fundamental laser beam acts as the “gate” light. After passing through the delay line, the “gate” beam sums to the sample emission promoting the up-conversion process. The time resolution is about 200 fs while the spectra resolution is 1.5 nm. All the measurements were carried out at ca. 2.0 × 10^−3^ J/cm^2^ of energy fluence and under the magic angle condition, stirring the solution in a 2 mm cuvette (0.5 < A < 1.0 at λ_exc_ = 400 nm) during the experiments to avoid photoproduct interferences. Photodegradation was checked by recording the absorption spectra before and after the time-resolved measurement, where no significant change was observed. The experimental data matrixes were first analyzed using the Surface Xplorer PRO (Ultrafast Systems) software, where it was possible to perform SVD of the 3D matrix to derive the principal components (spectra and kinetics). Successively, a global analysis using GloTarAn software was performed in order to obtain the lifetimes and the evolution-associated spectra (EAS) of the detected transient.

Triplet formation quantum yields, transient absorption, and triplet lifetimes were measured using nanosecond (pulse width 7 ns and laser energy < 1 mJ pulse^−1^) laser flash photolysis (Edinburgh LP980) with a pump pulse centered at 355 nm (third harmonic of a Continuum Surelite II Nd:YAG laser, Spectra Physics), coupled with a PMT for signal detection. A pulsed xenon lamp was then used to probe the absorption properties of the produced excited states. The setup was calibrated using an optically matched solution of Benzophenone in AcCN (Φ_T_ = 1.0 and ε_T_ = 5200 M^−1^ cm^−1^) [65]. Triplet–triplet absorption coefficients (ε_T_) were measured by energy transfer experiments from dithienylketone (DTK, Φ_T_ = 1.0 and ε_T_ = 5200 M^−1^ cm^−1^ at λ_T_ = 630 nm) to the DMA-QP and DMA-QF compounds, and from DMA-QF and DMA-QT to the all-trans-α, ω-di(2-thienyl)octatetraene (D2TO, Φ_T_ < 0.005 and ε_T_ = 53,000 M^−1^ cm^−1^ at 465 nm) [66]. The triplet quantum yields were determined by an actinometry approach considering Thioxanten-9-one (TX) in AcCN (Φ_T_ = 0.66 [67] and ε_T_ = 30,000 M^−1^ cm^−1^ at λ_T_ = 630 nm) [68] and Anthracene (A) in CH (Φ_T_ = 0.71 and ε_T_ = 45,500 M^−1^ cm^−1^ at λ_T_ = 422 nm) [65] as references (uncertainties ca. ±15% on Φ_T_). All measurements were performed by bubbling the sample with pure N_2_.

The same Nd:YAG pump coupled with an optical parametric oscillator (OPO-Surelite P/N 996-0210, Continuum, Newton, MA, USA) was used to perform the two-photon excited fluorescence experiments. The OPO can be tuned to produce radiation in the 410–2200 nm range (*signal* between 410 and 750 nm; *idler* between 820 and 2200 nm). The fluorescence intensity is detected by a PTM tube (Hamamatsu R2257-Y001), powered by a high-voltage power supply (PS-310, SRS) and given in mV by an oscilloscope LeCroy-Wave Runner-LT322 (500 MHz, 200 MS s^−1^, DSO). Solutions of known concentration (about 1 × 10^−5^ M) in DMF were prepared together with fluorescein in buffered water at pH 11 (σ = 26 GM at 930 nm) [69] as reference compounds to determine the TPA cross-sections through a comparative approach.

### 4.5. Quantum Mechanical Calculations

Quantum mechanical calculations were performed by using the Gaussian 16 package [70]. DFT with the B3LYP functional was chosen as the method to optimize the ground state geometry of these small organic push-pull systems and to derive their properties [71]. In contrast the lowest singlet excited states were investigated using TD–DFT excited-state calculations with WB97XD functional. Every calculation was submitted using 6–31 g + G(d) as the basis set including the solvent effect (Tol) according to the conductor-like polarizable continuum model (CPCM) [72].

## Figures and Tables

**Table 1:** Spectral properties of the investigated compounds in different solvents (dielectric constant, ε, and refractive index, *n*) absorption maximum (λ_abs_), emission maximum (λ_em_), Stokes shift ($\Delta \tilde{\nu }$). Bold values refer to the main absorption or emission maxima.

		DMA-QP		DMA-QF			DMA-QT			
Solvent	*f* (ε, *n*) ^a^	λ_abs_/nm	λ_em_/nm	$\Delta \tilde{\nu }/{\mathrm{cm}}^{-1}$	λ_abs_/nm	λ_em_/nm	$\Delta \tilde{\nu }/{\mathrm{cm}}^{-1}$	λ_abs_/nm	λ_em_/nm	$\Delta \tilde{\nu }/{\mathrm{cm}}^{-1}$
Tol	0.02423	368	430	3920	428	484	2700	**438**, 462 ^sh^	**488**, 523	1200
Tol/An (50/50 *v*/*v*)	0.14278	367	437	4365	433	504	3250	**440**, 462 ^sh^	**496**, 528	1480
An	0.22353	371	448	4630	435	523	3570	**442**, 462 ^sh^	**504**, 533	1800
EtOAc	0.36011	363	443	4975	431	527	4230	**430**, 455 ^sh^	496 ^sh^, **521**	1820
An/DCE (50/50 *v*/*v*)	0.39998	369	452	4980	436	540	4420	**440**, 462 ^sh^	504, **533**	1800
DCE	0.4968	368	458	5340	437	545	4540	**437**, 462 ^sh^	513 ^sh^, **532**	2850
DCE/DMF (50/50 *v*/*v*)	0.58324	368	468	5810	437	568	5280	**438**, 462 ^sh^	536	2990
EtOH	0.6668	367	480	6415	452	612	5780	**429**, 455 ^sh^	522	2820
AcCN	0.66697	364	451	5300	431	581	5990	**432**,455 ^sh^	537	3360
DMF	0.71215	368	474	6080	437	579	5610	**440**, 462 ^sh^	548	3400

^a^ $f\left(\varepsilon ,n\right)=\left(\frac{\varepsilon -1}{\varepsilon +2}-\frac{n^{2}-1}{n^{2}+2}\right)$; ^sh^ = shoulder.

**Table 2:** Predicted and experimental data required for the determination of the dynamic (β_CT_) and static (β_0_) hyperpolarizability coefficients of the investigated compounds in Tol: ground state dipole moment (μ_GS_); excited-state dipole moment (μ_ES_); Onsager’s cavity ray (*a*); dipole moment difference (Δμ_CT_ = μ_ES_ − μ_GS_); absorption maximum frequency ($\tilde{v_{eg}}$); theoretical oscillator strength (*f*). In the case of DMA-QT, the distance between the barycenter of the two poles (*d* = 8.00 Å) is also used as the double dipole model is considered.

	Calculated Parameter				Experimental Parameter				
Compound	μ_GS_/D	μ_ES_/D	*a*/Å	*f*	Slope	$\tilde{\nu_{eg}}/{\mathrm{cm}}^{-1}$	Δμ_CT_/D	β_CT_/10^−30^ esu^−1^ cm^5^	β_0_/10^−30^ esu^−1^ cm^5^
DMA-QP	3.41	6.68	11.1	1.3168	3200	27,170	20.9	248	114
DMA-QF	3.13	3.87	11.8	1.5571	4700	23,360	27.6	924	273
DMA-QT	1.56	2.40	12.9	1.9155	3200	22,830	21.1	1030	276

**Table 3:** Fs-TA and FUC results of global analysis of compounds DMA-QP, DAQF, and DMA-QT in Tol and DMF obtained by exciting at 400 nm.

	DMA-QP		DMA-QF		DMA-QT		
Solvent	τ/ps						Assignment
	TA	FUC	TA	FUC	TA	FUC	
Tol					0.90	0.39	Solv._i_.
	1.7	3.5	2.7	2.8	6.4	5.5	Solv._d_.
					280	130	SR
	88	89	110	93	900	840	S_1_
	Inf		Inf		Inf		T_1_
DMF	1.5	0.97	0.77	0.68	0.57	0.47	Solv._i_., S_1,LE_
			3.3	3.0	3.1	2.2	Solv._d_.
	150	140	280	270	170	110	SR
	1100	970	860	830	1000	840	S_1, ICT_
	Inf		Inf		Inf		T_1_

**Table 4:** Triplet properties of investigated compounds in solvents of different polarity: absorption maximum (λ_T_) and triplet lifetime in the presence (τ_T,AIR_) and absence (τ_T,N2_) of molecular oxygen with the relative quenching constant (k_OX_); molar absorption coefficient (ε_T_) and triplet quantum yield (Φ_T_), kinetic constant (given by k_T_ = Φ_T_/τ_S1_); singlet oxygen quantum yield (Φ_∆_).

Compound	Solvent	λ_T_/nm	τ_T,AIR_/μs	τ_T,N2_/μs	k_OX_/10^9^ s^−1^	ε_T_ */M^−1^ cm^−1^	Φ_T_	k_T_/10^8^ s^−1^	Φ_Δ_
DMA-QP	Tol	460	0.23	4.63	4.2	5900	0.11	12	
	DMF	450	0.09	3.14	0.28		<0.05		
DMA-QF	Tol	650	0.09	0.41	8.8	4500	0.41	4.9	
	DMF	600	0.13				<0.01		
DMA-QT	Tol	610	0.135	16.4	7.5	26,600	0.80	9.6	0.69
	DMF	610	0.22	14.8	11.5		0.43	5.1	0.73

* In CH.

## Data Availability

Data are contained within the article or Supplementary Material.

## Supplementary Materials

The following supporting information can be downloaded at https://www.mdpi.com/article/10.3390/molecules27248725/s1: Figure S1 and Tables S1 and S2: spectral and fluorescence properties; Figures S2–S5 and Tables S3–S18: quantum mechanical calculations; Figures S6–S10 and Table S19: femtosecond transient absorption and fluorescence up-conversion; Figures S11–S13: nanosecond transient absorption; Figure S14: singlet oxygen phosphorescence; Figures S15–S18: ^1^H and ^13^C NMR spectra (CDCl_3_) of DMA-QF and DMA-QT.

## Sample Availability

Samples of the compounds DMA-DP, DMA-QF, and DMA-QT are available from the authors.
