# Supplementary material for: Tuning the Photophysics of Two-Arm Bis[(dimethylamino)styryl]benzene Derivatives by Heterocyclic Substitution
Source: Molecules. 2022 Dec 9;27(24):8725. doi: 10.3390/molecules27248725 (PMC9787945; doi:10.3390/molecules27248725)
Supplement: Supplementary file 1 [file molecules-27-08725-s001.zip › molecules-2036337-supplementary.pdf]

Supplementing material

Tuning the Photophysics of Two-Arm Bis[(Dimethylamino)Styryl]Benzene Derivatives by Heterocyclic Substitution

Letizia Mencaroni [1], Alessio Cesaretti [1,*], Benedetta Carlotti [1], Martina Alebardi [1], Fausto Elisei [1], Ana Ratković [2,†], Irena Škorić [2] and Anna Spalletti [1]

[1] Department of Chemistry, Biology and Biotechnology and Center of Excellence on Innovative Nanostructured Materials (CEMIN), University of Perugia, Via Elce di Sotto n.8, 06123 Perugia, Italy
[2] Department of Organic Chemistry, Faculty of Chemical Engineering and Technology, University of Zagreb, HR-10 000 Zagreb, Croatia
* Correspondence: alex.cesaretti14@gmail.com
† Current address: Selvita d.o.o., Prilaz baruna Filipovića 29, HR-10 000 Zagreb, Croatia.

List of contents

1. *Spectral and fluorescence properties*
2. *Quantum mechanical calculations*
3. *Femtosecond transient absorption and fluorescence up conversion*
4. *Nanosecond transient absorption*
5. *Singlet oxygen phosphorescence*
6. *^{1}H and ^{13}C NMR spectra ($CDCl_3$) of **DMA-QT** and **DMA-QF***

1. *Spectral and fluorescence properties*

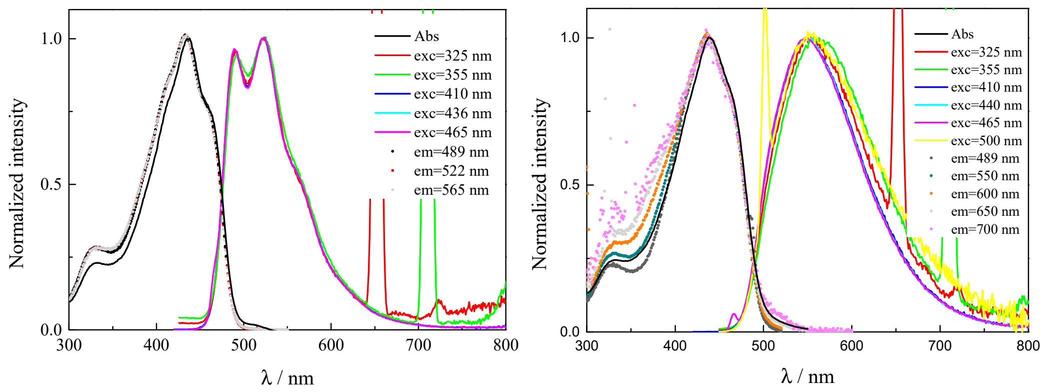

Figure S1: Wavelength effect on normalized emission and excitation spectra of compound **DMA-QT** in Tol (left) and DMF (right).

Table S1: Molar absorption coefficients (ε_G) in Toluene and fluorescence properties of DMA-derivatives in solvents of different polarity.

		**DMA-QP**	**DMA-QF**	**DMA-QT**
ε_G / $M^{-1}cm^{-1}$		34600	30200	28800
Solvent	ε	Φ_F	Φ_F	Φ_F
Tol	2.39	0.07	0.06	0.47
Tol/An (50/50 v/v)	3.36	0.09	0.08	0.49
An	4.33	0.06	0.08	0.50
EtOAc	6.05	0.05	0.07	0.48
An/DCE (50/50 v/v)	7.49	0.05	0.10	0.50
DCE	10.65	0.05	0.06	0.57
DCE/DMF (50/50 v/v)	24.4	0.10	0.12	0.55
EtOH	24.55	0.06	0.11	0.58
AcCN	37.5	0.20	0.37	0.53
DMF	38.2	0.12	0.20	0.52

Table S2: Fluorescence properties of the investigated molecules: fluorescence quantum yields (Φ_F), fluorescence lifetimes (τ_F), provided by fluorescence up conversion measurements, relative kinetic constants (k_F) and the non-radiative kinetic constants (k_{nr}).

		**DMA-QP**				**DMA-QF**				**DMA-QT**			
Solvent	ε	Φ_F	τ_F / ns	$k_F{}^{a}$ / 10^8 s^{-1}	$k_{nr}{}^{b}$ / 10^8 s^{-1}	Φ_F	τ_F / ns	$k_F{}^{a}$ / 10^8 s^{-1}	$k_{nr}{}^{b}$ / 10^8 s^{-1}	Φ_F	τ_F / ns	$k_F{}^{a}$ / 10^8 s^{-1}	$k_{nr}{}^{b}$ / 10^8 s^{-1}
Tol	2.39	0.07	0.089	7.9	92	0.06	0.093	6.5	57	0.47	0.84	6.1	-
DMF	38.2	0.12	0.97	1.2	9.1	0.20	0.83	2.3	9.6	0.52	0.84	6.2	0.60

[a] $k_F = \frac{\Phi_F}{\tau_F}$.

[b] $k_{nr} = \frac{1-\Phi_F-\Phi_T}{\tau_F}$.

2. *Quantum mechanical calculations*

Table S3: Optimized S_0 geometries and relative energies of the **compressed**, **semi-elongated** and **elongated** conformers of the DMA-derivatives calculated by B3LYP/6-31+G(d) model in Toluene (CPCM).

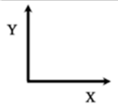		**compressed**	**semi-elongated**	**elongated**
**DMA-QP**	**ΔH_f / Ha**	0	0.001147	0.002432
	**ΔH_f / kcal mol^{-1}**	0	0.719	1.525
**DMA-QF**		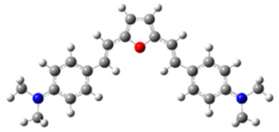	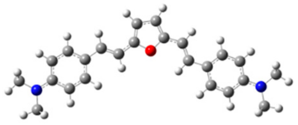	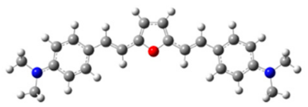
	**ΔH_f / Ha**	0	0.001924	0.003772
	**ΔH_f / kcal mol^{-1}**	0	1.206	2.435
**DMA-QT**	**ΔH_f / Ha**	0	0.002082	0.004097
	**ΔH_f / kcal mol^{-1}**	0	1.305	2.568

Table S4: Optimized S_0 (B3LYP/6-31+G(d)) geometries of the compressed conformers of the DMA-derivatives.

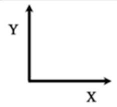		**S_0**
**DMA-QP**		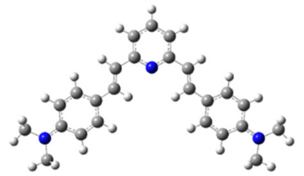
	N···H / Å	2.54
	N$\hat{H}$C / °	97.8
**DMA-QF**		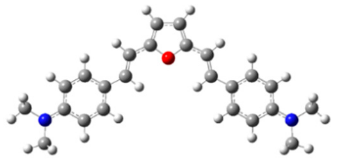
	O···H / Å	2.56
	O$\hat{H}$C / °	96.9
**DMA-QT**		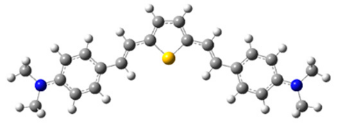
	S···H / Å	2.83
	S$\hat{H}$C / °	102.9

Table S5: Dipole (μ / D, Mulliken charges) and quadrupole moments (field-independent basis, D×Å) of the ground and the lowest excited singlet state (in the Franck-Condon, FC, and optimized, rel, structure) of DMA-derivatives in Toluene (CPCM) calculated by the WB97XD/6-31+G(d) model.

Compound	Conformer	State	$\|\mu_x\|$	$\|\mu_Y\|$	$\|\mu_Z\|$	$\|\mu\|$	Q_{XX}	Q_{YY}	Q_{ZZ}	Q_{XY}	Q_{XZ}	Q_{YZ}
**DMA-QP**	**compr**	**S_0**	**0.00**	**3.41**	**0.00**	**3.41**	**-77**	**-129**	**-181**	**0**	**0**	**0**
		$S_{1,FC}$	0.00	4.15	0.00	4.15	-70	-130	-180	0	0	0
		**$S_{1,rel}$**	**0.00**	**6.68**	**0.00**	**6.68**	**-48**	**-132**	**-181**	**0**	**0**	**0**
	semielong	S_0	0.12	3.46	0.00	3.46	-56	-133	-181	0	0	0
		$S_{1,FC}$	0.17	3.35	0.00	3.36	-57	-132	-180	0	0	0
		$S_{1,rel}$	5.04	5.70	0.00	7.61	-29	-135	-181	-8	0	0
	elong	S_0	0.00	1.77	0.00	1.77	-54	-134	-181	0	0	0
		$S_{1,FC}$	0.00	2.47	0	2.47	-44	-135	-180	0	0	0
		$S_{1,rel}$	0.00	4.44	0.00	4.44	-3	-136	-181	0	0	0
**DMA-QF**	**compr**	**S_0**	**0.00**	**3.13**	**0.01**	**3.13**	**-64**	**-134**	**-175**	**0**	**0**	**0**
		$S_{1,FC}$	0.00	2.41	0.00	2.41	-62	-130	-174	0	0	0
		**$S_{1,rel}$**	**0.00**	**3.87**	**0.00**	**3.87**	**-53**	**-132**	**-175**	**0**	**0**	**0**
	semielong	S_0	0.03	1.90	0.00	1.90	-53	-133	-174	0	0	0
		$S_{1,FC}$	0.16	1.87	0.00	1.88	-54	-134	-181	0	0	0
		$S_{1,rel}$	0.34	2.85	0.00	2.87	-38	-135	-175	-4	0	0
	elong	S_0	0.00	1.94	0.00	1.94	-45	-138	-174	0	0	0
		$S_{1,FC}$	0.00	1.31	0.00	1.31	-47	-137	-174	0	0	0
		$S_{1,rel}$	0.00	1.79	0.00	1.79	-26	-138	-175	0	0	0
**DMA-QT**	**compr**	**S_0**	**0.00**	**1.56**	**0.00**	**1.56**	**-50**	**-145**	**-183**	**0**	**0**	**0**
		$S_{1,FC}$	0.00	1.13	0.00	1.13	-43	-144	-183	0	0	0
		**$S_{1,rel}$**	**0.00**	**2.40**	**0.00**	**2.40**	**-25**	**-145**	**-183**	**0**	**0**	**0**
	semielong	S_0	0.28	1.03	0.00	1.07	-45	-147	-183	0	0	0
		$S_{1,FC}$	0.08	0.56	0.00	0.56	-38	-147	-183	0	0	0
		$S_{1,rel}$	0.05	1.39	0.00	1.39	-17	-148	-183	0	0	0
	elong	S_0	0.00	0.56	0.00	0.56	-41	-149	-183	0	0	0
		$S_{1,FC}$	0.00	0.00	0.00	0.00	-34	-150	-183	0	0	0
		$S_{1,rel}$	0.00	0.40	0.00	0.40	-12	-150	-183	0	0	0

Table S6: Absorption and emission wavelengths (λ), oscillator strength (f) and molecular orbitals of the **compressed DMA-QP** conformer in Toluene (CPCM) calculated by the WB97XD/6-31+G(d)//B3LYP/6-31+G(d) model, together with the experimental absorption and emission maxima.

Transition	λ_{th}/nm	f	MOs	%	λ_{exp}/nm
$S_0 \rightarrow T_1$	591	0.0000	$\pi_H \rightarrow \pi^*_{L+1}$	48	
$S_0 \rightarrow T_2$	546	0.0000	$\pi_H \rightarrow \pi^*_L$	51	
$S_0 \rightarrow S_1$	368	1.3168	$\pi_H \rightarrow \pi^*_L$	72	367
$S_0 \rightarrow T_3$	362	0.0000	$\pi_{H-2} \rightarrow \pi^*_{L+1}$	29	
$S_0 \rightarrow T_4$	351	0.0000	$\pi_{H-2} \rightarrow \pi^*_L$	32	
$S_0 \rightarrow T_5$	335	0.0000	$\pi_{H-1} \rightarrow \pi^*_{L+3}$<br>$\pi_H \rightarrow \pi^*_{L+2}$	43<br>41	
$S_0 \rightarrow T_6$	334	0.0000	$\pi_{H-1} \rightarrow \pi^*_{L+2}$<br>$\pi_H \rightarrow \pi^*_{L+3}$	42<br>41	
$S_0 \rightarrow S_2$	331	1.1002	$\pi_{H-1} \rightarrow \pi^*_L$<br>$\pi_H \rightarrow \pi^*_{L+1}$	39<br>51	
$S_0 \rightarrow T_7$	313	0.0000	$\pi_{H-3} \rightarrow \pi^*_L$<br>$\pi_H \rightarrow \pi^*_{L+1}$	26<br>21	
$S_0 \rightarrow T_8$	308	0.0000	$\pi_{H-6} \rightarrow \pi^*_L$	83	
$S_0 \rightarrow T_9$	293	0.0000	$\pi_{H-3} \rightarrow \pi^*_{L+1}$	21	
$S_0 \rightarrow S_3$	288	0.1985	$\pi_{H-1} \rightarrow \pi^*_{L+1}$	49	
$S_0 \rightarrow T_{10}$	284	0.0000	$\pi_{H-5} \rightarrow \pi^*_{L+2}$<br>$n_{H-4} \rightarrow \pi^*_{L+3}$	36<br>37	
$S_0 \rightarrow S_4$	280	0.2872	$\pi_{H-1} \rightarrow \pi^*_{L+2}$<br>$\pi_H \rightarrow \pi^*_{L+3}$	37<br>42	
$S_0 \rightarrow S_5$	280	0.0114	$\pi_{H-1} \rightarrow \pi^*_{L+3}$<br>$\pi_H \rightarrow \pi^*_{L+2}$	38<br>37	
$S_0 \rightarrow S_6$	271	0.0033	$\pi_{H-6} \rightarrow \pi^*_L$	88	
$S_0 \rightarrow S_7$	270	0.0145	$\pi_{H-1} \rightarrow \pi^*_L$	48	
$S_0 \rightarrow S_8$	247	0.0000	$\pi_{H-6} \rightarrow \pi^*_{L+1}$	87	
$S_0 \rightarrow S_9$	238	0.0000	$\pi_{H-1} \rightarrow \pi^*_{L+5}$<br>$\pi_H \rightarrow \pi^*_{L+4}$	33<br>39	
$S_0 \rightarrow S_{10}$	237	0.0775	$\pi_{H-1} \rightarrow \pi^*_{L+4}$<br>$\pi_H \rightarrow \pi^*_{L+5}$	35<br>35	
$S_1 \rightarrow S_0$	404	1.2783	$\pi_H \rightarrow \pi^*_L$	77	430

Table S7: Molecular orbitals of **DMA-QP** in Tol.

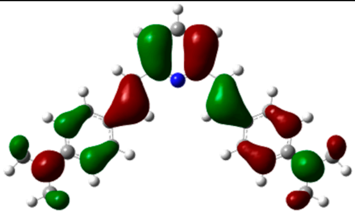	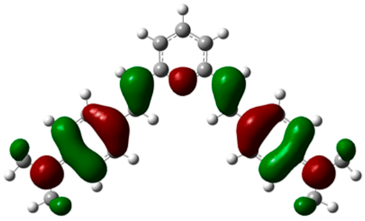	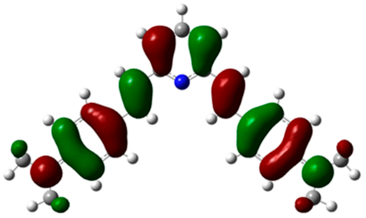
π_{H-2}	π_{H-1}	π_{H}
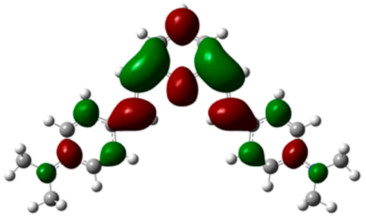	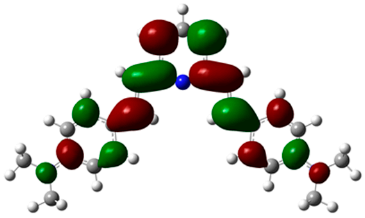	
π_{L}	π_{L+1}	

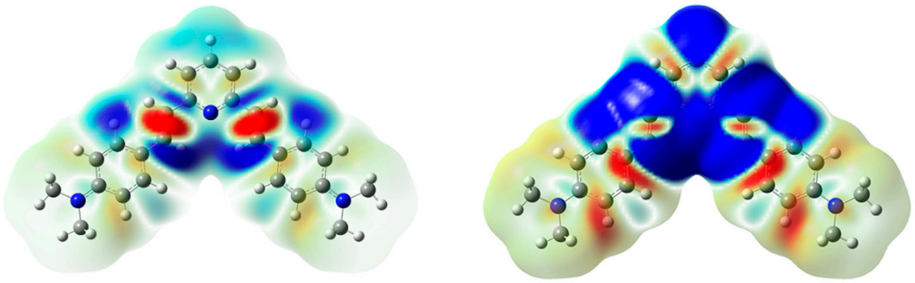

Figure S2: Effect of the $S_0 \rightarrow S_1$ (left) and $S_0 \rightarrow S_{1,rel}$ (right) transitions on the electron density of **DMA-QP**; increase and decrease of the electron densities are represented by blue (+0.00001) and red (-0.00001), respectively.

Table S8: Absorption and emission wavelengths (λ), oscillator strength (f) and molecular orbitals of **semi-elongated DMA-QP** conformer in Toluene (CPCM) calculated by the WB97XD/6-31+G(d)//B3LYP/6-31+G(d) model, together with the experimental absorption and emission maxima.

Transition	λ_{th}/nm	f	MOs	%	λ_{exp}/nm
$S_0 \rightarrow T_1$	600	0.0000	$\pi_H \rightarrow \pi^*_L$	51	
$S_0 \rightarrow T_2$	537	0.0000	$\pi_{H-1} \rightarrow \pi^*_L$<br>$\pi_H \rightarrow \pi^*_{L+1}$	30<br>46	
$S_0 \rightarrow T_3$	366	0.0000	$\pi_{H-2} \rightarrow \pi^*_L$	29	
$S_0 \rightarrow S_1$	356	1.8679	$\pi_H \rightarrow \pi^*_{L+1}$	63	367
$S_0 \rightarrow T_4$	343	0.0000	$\pi_{H-2} \rightarrow \pi^*_{L+1}$	31	
$S_0 \rightarrow S_2$	335	0.7830	$\pi_H \rightarrow \pi^*_L$	64	
$S_0 \rightarrow T_5$	335	0.0000	$\pi_H \rightarrow \pi^*_{L+2}$	45	
$S_0 \rightarrow T_6$	335	0.0000	$\pi_{H-1} \rightarrow \pi^*_{L+3}$<br>$\pi_H \rightarrow \pi^*_{L+3}$	35<br>47	
$S_0 \rightarrow T_7$	302	0.0000	$\pi_{H-3} \rightarrow \pi^*_{L+1}$<br>$\pi_{H-1} \rightarrow \pi^*_{L+1}$	25<br>19	
$S_0 \rightarrow T_8$	299	0.0000	$\pi_{H-3} \rightarrow \pi^*_L$	18	
$S_0 \rightarrow T_9$	296	0.0000	$\pi_{H-6} \rightarrow \pi^*_{L+1}$	78	
$S_0 \rightarrow S_3$	289	0.0349	$\pi_{H-1} \rightarrow \pi^*_L$	39	
$S_0 \rightarrow T_{10}$	283	0.0000	$\pi_{H-4} \rightarrow \pi^*_{L+3}$	52	
$S_0 \rightarrow S_4$	280	0.0856	$\pi_H \rightarrow \pi^*_{L+3}$	48	
$S_0 \rightarrow S_5$	279	0.1769	$\pi_{H-1} \rightarrow \pi^*_{L+2}$	36	
$S_0 \rightarrow S_6$	279	0.0298	$\pi_{H-1} \rightarrow \pi^*_{L+1}$	56	
$S_0 \rightarrow S_7$	250	0.0033	$\pi_{H-6} \rightarrow \pi^*_{L+1}$	76	
$S_0 \rightarrow S_8$	250	0.0001	$\pi_{H-6} \rightarrow \pi^*_L$	80	
$S_0 \rightarrow S_9$	238	0.0271	$\pi_{H-1} \rightarrow \pi^*_{L+5}$<br>$\pi_H \rightarrow \pi^*_{L+5}$	24<br>23	
$S_0 \rightarrow S_{10}$	237	0.0505	$\pi_{H-1} \rightarrow \pi^*_{L+4}$	33	
$S_1 \rightarrow S_0$	379	2.4277	$\pi_{H-1} \rightarrow \pi^*_L$<br>$\pi_H \rightarrow \pi^*_{L+1}$	39<br>55	430

Table S9: Absorption and emission wavelengths (λ), oscillator strength (f) and molecular orbitals of the **elongated DMA-QP** conformer in Toluene (CPCM) calculated by the WB97XD/6-31+G(d)//B3LYP/6-31+G(d) model, together with the experimental absorption and emission maxima.

Transition	λ_{th}/nm	f	MOs	%	λ_{exp}/nm
**$S_0 \rightarrow T_1$**	610	0.0000	$\pi_H \rightarrow \pi^*_L$	55	
**$S_0 \rightarrow T_2$**	531	0.0000	$\pi_{H-1} \rightarrow \pi^*_L$<br>$\pi_H \rightarrow \pi^*_{L+1}$	34<br>42	
**$S_0 \rightarrow T_3$**	369	0.0000	$\pi_{H-2} \rightarrow \pi^*_L$	29	
**$S_0 \rightarrow S_1$**	349	2.3852	$\pi_{H-1} \rightarrow \pi^*_L$<br>$\pi_H \rightarrow \pi^*_{L+1}$	40<br>51	367
**$S_0 \rightarrow S_2$**	341	0.4602	$\pi_H \rightarrow \pi^*_L$	73	
**$S_0 \rightarrow T_4$**	337	0.0000	$\pi_{H-1} \rightarrow \pi^*_{L+3}$<br>$\pi_H \rightarrow \pi^*_{L+2}$	32<br>39	
**$S_0 \rightarrow T_5$**	335	0.0000	$\pi_{H-1} \rightarrow \pi^*_{L+2}$<br>$\pi_H \rightarrow \pi^*_{L+3}$	32<br>44	
**$S_0 \rightarrow T_6$**	332	0.0000	$\pi_{H-2} \rightarrow \pi^*_{L+1}$<br>$\pi_H \rightarrow \pi^*_{L+6}$	26<br>27	
**$S_0 \rightarrow T_7$**	303	0.0000	$\pi_{H-3} \rightarrow \pi^*_L$<br>$\pi_H \rightarrow \pi^*_{L+1}$	17<br>15	
**$S_0 \rightarrow T_8$**	292	0.0000	$\pi_{H-3} \rightarrow \pi^*_{L+1}$<br>$\pi_{H-1} \rightarrow \pi^*_{L+1}$	25<br>19	
**$S_0 \rightarrow S_3$**	287	0.0204	$\pi_{H-1} \rightarrow \pi^*_L$<br>$\pi_H \rightarrow \pi^*_{L+1}$	25<br>22	
**$S_0 \rightarrow T_9$**	283	0.0000	$\pi_{H-5} \rightarrow \pi^*_{L+2}$<br>$\pi_{H-4} \rightarrow \pi^*_{L+3}$	27<br>34	
**$S_0 \rightarrow T_{10}$**	282	0.0000	$\pi_{H-5} \rightarrow \pi^*_{L+3}$<br>$\pi_{H-4} \rightarrow \pi^*_{L+2}$	34<br>27	
**$S_0 \rightarrow S_4$**	280	0.0336	$\pi_{H-1} \rightarrow \pi^*_{L+2}$<br>$\pi_H \rightarrow \pi^*_{L+3}$	31<br>43	
**$S_0 \rightarrow S_5$**	279	0.1780	$\pi_{H-1} \rightarrow \pi^*_{L+3}$	36	
**$S_0 \rightarrow S_6$**	262	0.0116	$\pi_{H-1} \rightarrow \pi^*_{L+1}$	55	
**$S_0 \rightarrow S_7$**	252	0.0000	$\pi_{H-6} \rightarrow \pi^*_L$	90	
**$S_0 \rightarrow S_8$**	247	0.0032	$\pi_{H-6} \rightarrow \pi^*_{L+1}$	78	
**$S_0 \rightarrow S_9$**	237	0.0000	$\pi_{H-1} \rightarrow \pi^*_{L+5}$<br>$\pi_H \rightarrow \pi^*_{L+4}$	35<br>37	
**$S_0 \rightarrow S_{10}$**	237	0.0774	$\pi_{H-1} \rightarrow \pi^*_{L+4}$<br>$\pi_H \rightarrow \pi^*_{L+5}$	35<br>36	
**$S_1 \rightarrow S_0$**	506	2.3488	$\pi_H \rightarrow \pi^*_L$	93	430

Table S10: Absorption and emission wavelengths (λ), oscillator strength (f) and molecular orbitals of **compressed DMA-QF** conformer in Toluene (CPCM) calculated by the WB97XD/6-31+G(d)//B3LYP/6-31+G(d) model, together with the experimental absorption and emission maxima.

Transition	λ_{th}/nm	f	MOs	%	λ_{exp}/nm
$S_0 \rightarrow T_1$	826	0.0000	$\pi_H \rightarrow \pi^*_L$	82	
$S_0 \rightarrow T_2$	522	0.0000	$\pi_{H-1} \rightarrow \pi^*_L$<br>$\pi_H \rightarrow \pi^*_{L+1}$	34<br>47	
$S_0 \rightarrow S_1$	426	1.5571	$\pi_H \rightarrow \pi^*_L$	90	428
$S_0 \rightarrow T_3$	377	0.0000	$\pi_{H-2} \rightarrow \pi^*_L$<br>$\pi_{H-1} \rightarrow \pi^*_{L+1}$	30<br>27	
$S_0 \rightarrow T_4$	342	0.0000	$\pi_{H-1} \rightarrow \pi^*_{L+2}$<br>$\pi_H \rightarrow \pi^*_{L+3}$	39<br>47	
$S_0 \rightarrow T_5$	342	0.0000	$\pi_{H-1} \rightarrow \pi^*_{L+3}$<br>$\pi_H \rightarrow \pi^*_{L+2}$	39<br>47	
$S_0 \rightarrow T_6$	325	0.0000	$\pi_{H-2} \rightarrow \pi^*_{L+2}$	26	
$S_0 \rightarrow S_2$	317	0.6908	$\pi_{H-1} \rightarrow \pi^*_L$<br>$\pi_H \rightarrow \pi^*_{L+1}$	48<br>43	
$S_0 \rightarrow T_7$	287	0.0000	$\pi_{H-4} \rightarrow \pi^*_{L+3}$<br>$\pi_{H-3} \rightarrow \pi^*_{L+2}$	27<br>30	
$S_0 \rightarrow S_3$	287	0.0051	$\pi_H \rightarrow \pi^*_{L+2}$	51	
$S_0 \rightarrow T_8$	284	0.0000	$\pi_{H-4} \rightarrow \pi^*_{L+2}$<br>$\pi_{H-3} \rightarrow \pi^*_{L+3}$	38<br>35	
$S_0 \rightarrow S_4$	284	0.3700	$\pi_H \rightarrow \pi^*_{L+3}$	47	
$S_0 \rightarrow T_9$	270	0.0000	$\pi_H \rightarrow \pi^*_{L+1}$	26	
$S_0 \rightarrow S_5$	269	0.0726	$\pi_H \rightarrow \pi^*_{L+1}$	47	
$S_0 \rightarrow T_{10}$	263	0.0000	$\pi_{H-11} \rightarrow \pi^*_L$<br>$\pi_{H-5} \rightarrow \pi^*_{L+1}$	15<br>17	
$S_0 \rightarrow S_6$	257	0.1547	$\pi_{H-2} \rightarrow \pi^*_L$	53	
$S_0 \rightarrow S_7$	248	0.0000	$\pi_H \rightarrow \pi^*_{L+4}$	46	
$S_0 \rightarrow S_8$	242	0.0627	$\pi_H \rightarrow \pi^*_{L+5}$	41	
$S_0 \rightarrow S_9$	237	0.0000	$\pi_H \rightarrow \pi^*_{L+6}$	36	
$S_0 \rightarrow S_{10}$	229	0.0000	$\pi_H \rightarrow \pi^*_{L+7}$	67	
$S_1 \rightarrow S_0$	519	1.4944	$\pi_H \rightarrow \pi^*_L$	94	484

Table S11: Molecular orbitals of the **compressed DMA-QF** conformer in Tol.

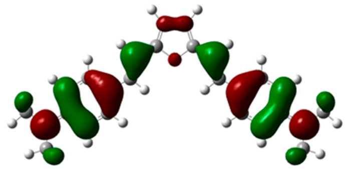	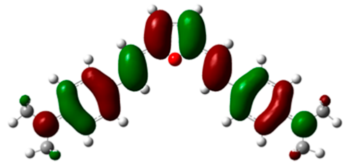
π_{H-1}	π_H
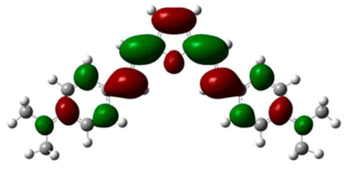	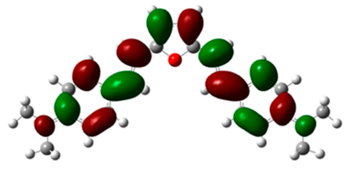
π_L	π_{L+1}

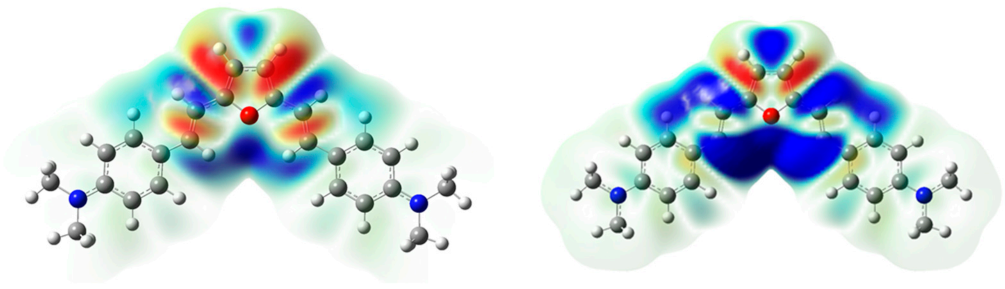

Figure S3: Effect of the $S_0 \rightarrow S_1$ (left) and $S_0 \rightarrow S_{1,rel}$ (right) transitions on the electron density of **DMA-QF (compressed)**; increase and decrease of the electron densities are represented by blue (+0.00001) and red (-0.00001), respectively.

Table S12: Absorption and emission wavelengths (λ), oscillator strength (f) and molecular orbitals of the **semi-elongated DMA-QF** conformer in Toluene (CPCM) calculated by the WB97XD/6-31+G(d)//B3LYP/6-31+G(d) model, together with the experimental absorption and emission maxima.

Transition	λ_{th}/nm	f	MOs	%	λ_{exp}/nm
**$S_0 \rightarrow T_1$**	819	0.0000	$\pi_H \rightarrow \pi^*_L$	81	
**$S_0 \rightarrow T_2$**	531	0.0000	$\pi_H \rightarrow \pi^*_{L+1}$	50	
**$S_0 \rightarrow S_1$**	421	1.9880	$\pi_H \rightarrow \pi^*_L$	89	428
**$S_0 \rightarrow T_3$**	377	0.0000	$\pi_{H-2} \rightarrow \pi^*_L$<br>$\pi_{H-1} \rightarrow \pi^*_{L+1}$	30<br>26	
**$S_0 \rightarrow T_4$**	342	0.0000	$\pi_H \rightarrow \pi^*_{L+3}$	45	
**$S_0 \rightarrow T_5$**	342	0.0000	$\pi_{H-1} \rightarrow \pi^*_{L+2}$<br>$\pi_H \rightarrow \pi^*_{L+2}$	26<br>43	
**$S_0 \rightarrow T_6$**	331	0.0000	$\pi_{H-2} \rightarrow \pi^*_{L+1}$	26	
**$S_0 \rightarrow S_2$**	320	0.4903	$\pi_{H-1} \rightarrow \pi^*_L$<br>$\pi_H \rightarrow \pi^*_{L+1}$	38<br>53	
**$S_0 \rightarrow S_3$**	287	0.1168	$\pi_{H-1} \rightarrow \pi^*_{L+3}$<br>$\pi_H \rightarrow \pi^*_{L+2}$	27<br>45	
**$S_0 \rightarrow T_7$**	286	0.0000	$\pi_{H-4} \rightarrow \pi^*_{L+2}$<br>$\pi_{H-3} \rightarrow \pi^*_{L+3}$	30<br>21	
**$S_0 \rightarrow T_8$**	284	0.0000	$\pi_{H-4} \rightarrow \pi^*_{L+2}$<br>$\pi_{H-3} \rightarrow \pi^*_{L+3}$	26<br>33	
**$S_0 \rightarrow S_4$**	284	0.0955	$\pi_H \rightarrow \pi^*_{L+3}$	43	
**$S_0 \rightarrow S_5$**	271	0.0128	$\pi_{H-1} \rightarrow \pi^*_L$<br>$\pi_H \rightarrow \pi^*_{L+1}$	40<br>35	
**$S_0 \rightarrow T_9$**	268	0.0000	$\pi_{H-5} \rightarrow \pi^*_L$<br>$\pi_H \rightarrow \pi^*_{L+1}$	17<br>24	
**$S_0 \rightarrow T_{10}$**	265	0.0000	$n_{H-11} \rightarrow \pi^*_L$<br>$\pi_{H-5} \rightarrow \pi^*_{L+1}$	14<br>16	
**$S_0 \rightarrow S_6$**	256	0.1422	$\pi_{H-2} \rightarrow \pi^*_L$	51	
**$S_0 \rightarrow S_7$**	247	0.0008	$\pi_H \rightarrow \pi^*_{L+4}$	38	
**$S_0 \rightarrow S_8$**	242	0.0608	$\pi_H \rightarrow \pi^*_{L+5}$	38	
**$S_0 \rightarrow S_9$**	236	0.0000	$\pi_H \rightarrow \pi^*_{L+6}$	39	
**$S_0 \rightarrow S_{10}$**	228	0.0007	$\pi_H \rightarrow \pi^*_{L+7}$	59	
**$S_1 \rightarrow S_0$**	512	1.9912	$\pi_H \rightarrow \pi^*_L$	93	484

Table S13: Absorption and emission wavelengths (λ), oscillator strength (f) and molecular orbitals of **elongated DMA-QF** conformer in Toluene (CPCM) calculated by the WB97XD/6-31+G(d)//B3LYP/6-31+G(d) model, together with the experimental absorption and emission maxima.

Transition	λ_{th}/nm	f	MOs	%	λ_{exp}/nm
$S_0 \rightarrow T_1$	810	0.0000	$\pi_H \rightarrow \pi^*_L$	81	
$S_0 \rightarrow T_2$	541	0.0000	$\pi_H \rightarrow \pi^*_{L+1}$	53	
$S_0 \rightarrow S_1$	418	2.3217	$\pi_H \rightarrow \pi^*_L$	89	428
$S_0 \rightarrow T_3$	377	0.0000	$\pi_{H-2} \rightarrow \pi^*_L$<br>$\pi_{H-1} \rightarrow \pi^*_{L+1}$	30<br>25	
$S_0 \rightarrow T_4$	342	0.0000	$\pi_{H-1} \rightarrow \pi^*_{L+2}$<br>$\pi_H \rightarrow \pi^*_{L+3}$	39<br>46	
$S_0 \rightarrow T_5$	342	0.0000	$\pi_{H-1} \rightarrow \pi^*_{L+3}$<br>$\pi_H \rightarrow \pi^*_{L+2}$	39<br>46	
$S_0 \rightarrow T_6$	337	0.0000	$\pi_{H-2} \rightarrow \pi^*_{L+1}$	27	
$S_0 \rightarrow S_2$	326	0.2622	$\pi_H \rightarrow \pi^*_{L+1}$	62	
$S_0 \rightarrow S_3$	287	0.0779	$\pi_H \rightarrow \pi^*_{L+2}$	50	
$S_0 \rightarrow T_7$	286	0.0000	$\pi_{H-4} \rightarrow \pi^*_{L+3}$<br>$\pi_{H-3} \rightarrow \pi^*_{L+2}$	31<br>34	
$S_0 \rightarrow T_8$	284	0.0000	$\pi_{H-4} \rightarrow \pi^*_{L+2}$<br>$\pi_{H-3} \rightarrow \pi^*_{L+3}$	39<br>35	
$S_0 \rightarrow S_4$	284	0.0201	$\pi_H \rightarrow \pi^*_{L+3}$	49	
$S_0 \rightarrow S_5$	272	0.0070	$\pi_{H-1} \rightarrow \pi^*_L$	49	
$S_0 \rightarrow T_9$	268	0.0000	$\pi_{H-11} \rightarrow \pi^*_L$<br>$\pi_{H-5} \rightarrow \pi^*_{L+1}$	13<br>16	
$S_0 \rightarrow T_{10}$	266	0.0000	$\pi_{H-5} \rightarrow \pi^*_L$<br>$\pi_H \rightarrow \pi^*_{L+1}$	18<br>22	
$S_0 \rightarrow S_6$	256	0.1510	$\pi_{H-2} \rightarrow \pi^*_L$	51	
$S_0 \rightarrow S_7$	245	0.0000	$\pi_H \rightarrow \pi^*_{L+4}$	40	
$S_0 \rightarrow S_8$	242	0.0623	$\pi_H \rightarrow \pi^*_{L+5}$	41	
$S_0 \rightarrow S_9$	236	0.0000	$\pi_H \rightarrow \pi^*_{L+6}$	42	
$S_0 \rightarrow S_{10}$	227	0.0000	$\pi_H \rightarrow \pi^*_{L+7}$	52	
$S_1 \rightarrow S_0$	506	2.3488	$\pi_H \rightarrow \pi^*_L$	93	484

Table S14: Molecular orbitals of **elongated DMA-QF** conformer in Tol.

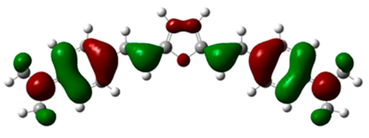	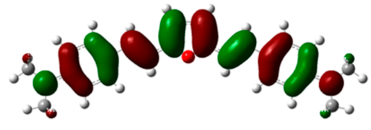
π_{H-1}	π_H
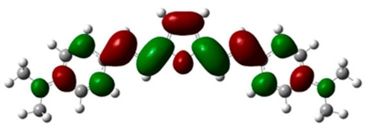	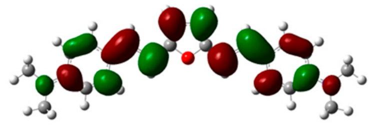
π_L	π_{L+1}

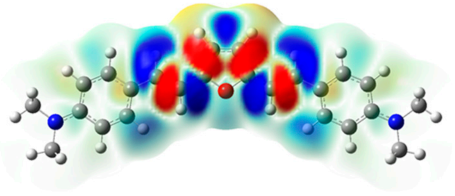

Figure S4: Effect of the $S_0 \rightarrow S_1$ transition on the electron density of **elongated DMA-QF** conformer; increase and decrease of the electron densities are represented by blue (+0.00001) and (-0.00001), respectively.

Table S15: Absorption and emission wavelengths (λ), oscillator strength (f) and molecular orbitals of **compressed DMA-QT** conformer in Toluene (CPCM) calculated by the WB97XD/6-31+G(d)//B3LYP/6-31+G(d) model, together with the experimental absorption and emission maxima.

**Transition**	**λ_{th}/nm**	**f**	**MOs**	**%**	**λ_{exp}/nm**
$S_0 \rightarrow T_1$	883	0.0000	$\pi_H \rightarrow \pi^*_L$	82	
$S_0 \rightarrow T_2$	523	0.0000	$\pi_{H-1} \rightarrow \pi^*_L$<br>$\pi_H \rightarrow \pi^*_{L+1}$	36<br>42	
$S_0 \rightarrow S_1$	438	1.9155	$\pi_H \rightarrow \pi^*_L$	89	436
$S_0 \rightarrow T_3$	388	0.0000	$\pi_{H-2} \rightarrow \pi^*_L$<br>$\pi_{H-1} \rightarrow \pi^*_{L+1}$	26<br>27	
$S_0 \rightarrow T_4$	341	0.0000	$\pi_{H-1} \rightarrow \pi^*_{L+2}$<br>$\pi_H \rightarrow \pi^*_{L+3}$	40<br>46	
$S_0 \rightarrow T_5$	341	0.0000	$\pi_{H-1} \rightarrow \pi^*_{L+3}$<br>$\pi_H \rightarrow \pi^*_{L+2}$	39<br>46	
$S_0 \rightarrow S_2$	323	0.2536	$\pi_{H-1} \rightarrow \pi^*_L$	60	
$S_0 \rightarrow T_6$	322	0.0000	$\pi_{H-2} \rightarrow \pi^*_{L+1}$	22	
$S_0 \rightarrow T_7$	306	0.0000	$\pi_{H-3} \rightarrow \pi^*_L$	74	
$S_0 \rightarrow T_8$	287	0.0000	$\pi_{H-5} \rightarrow \pi^*_{L+2}$<br>$\pi_{H-1} \rightarrow \pi^*_L$	26<br>24	
$S_0 \rightarrow S_3$	287	0.0035	$\pi_H \rightarrow \pi^*_{L+2}$	50	
$S_0 \rightarrow T_9$	287	0.0000	$\pi_{H-5} \rightarrow \pi^*_{L+3}$<br>$\pi_{H-4} \rightarrow \pi^*_{L+2}$	33<br>39	
$S_0 \rightarrow S_4$	285	0.1872	$\pi_{H-1} \rightarrow \pi^*_{L+2}$<br>$\pi_H \rightarrow \pi^*_{L+3}$	32<br>43	
$S_0 \rightarrow T_{10}$	273	0.0000	$\sigma_{H-11} \rightarrow \pi^*_L$<br>$\pi_{H-6} \rightarrow \pi^*_{L+1}$	13<br>16	
$S_0 \rightarrow S_5$	272	0.0431	$\pi_H \rightarrow \pi^*_{L+1}$	46	
$S_0 \rightarrow S_6$	270	0.3016	$\pi_{H-2} \rightarrow \pi^*_L$	48	
$S_0 \rightarrow S_7$	269	0.1764	$\pi_{H-3} \rightarrow \pi^*_L$	77	
$S_0 \rightarrow S_8$	263	0.0019	$\pi_H \rightarrow \pi^*_{L+12}$	39	
$S_0 \rightarrow S_9$	261	0.0000	$\pi_H \rightarrow \pi^*_{L+4}$	52	
$S_0 \rightarrow S_{10}$	255	0.0624	$\pi_H \rightarrow \pi^*_{L+5}$	40	
$S_1 \rightarrow S_0$	540	1.8379	$\pi_H \rightarrow \pi^*_L$	93	488

Table S16: Molecular orbitals of **compressed DMA-QT** conformer in Tol.

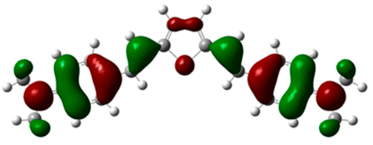	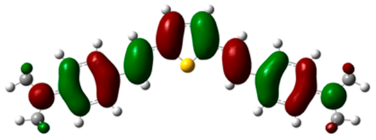
π_{H-1}	π_{H}
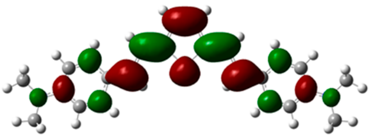	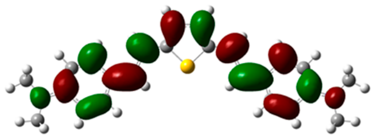
π_{L}	π_{L+1}

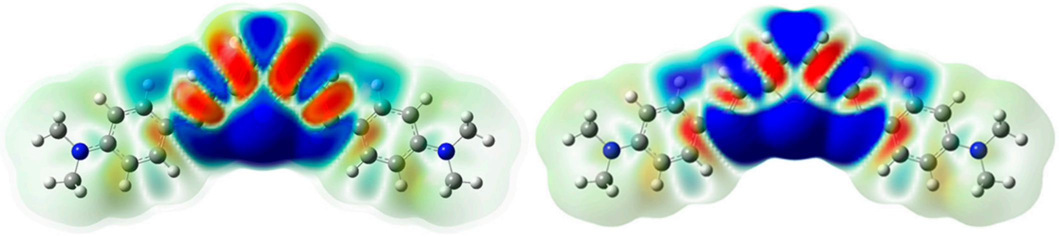

Figure S5: Effect of the $S_0 \rightarrow S_1$ (left) and $S_0 \rightarrow S_{1,rel}$ (right) transitions on the electron density of **DMA-QT**; increase and decrease of the electron densities are represented by blue (+0.00001) and (-0.00001), respectively.

Table S17: Absorption and emission wavelengths (λ), oscillator strength (f) and molecular orbitals of the **semi-elongated DMA-QT** conformer in Toluene (CPCM) calculated by the WB97XD/6-31+G(d)//B3LYP/6-31+G(d) model, together with the experimental absorption and emission maxima.

Transition	λ_{th}/nm	f	MOs	%	λ_{exp}/nm
$S_0 \rightarrow T_1$	879	0.0000	$\pi_H \rightarrow \pi^*_L$	81	
$S_0 \rightarrow T_2$	531	0.0000	$\pi_{H-1} \rightarrow \pi^*_L$<br>$\pi_H \rightarrow \pi^*_{L+1}$	34<br>45	
$S_0 \rightarrow S_1$	435	2.1737	$\pi_H \rightarrow \pi^*_L$	89	436
$S_0 \rightarrow T_3$	388	0.0000	$\pi_{H-2} \rightarrow \pi^*_L$<br>$\pi_{H-1} \rightarrow \pi^*_{L+1}$	26<br>26	
$S_0 \rightarrow T_4$	342	0.0000	$\pi_H \rightarrow \pi^*_{L+3}$	34	
$S_0 \rightarrow T_5$	341	0.0000	$\pi_{H-1} \rightarrow \pi^*_{L+2}$<br>$\pi_H \rightarrow \pi^*_{L+2}$	22<br>31	
$S_0 \rightarrow T_6$	327	0.0000	$\pi_{H-2} \rightarrow \pi^*_{L+1}$	24	
$S_0 \rightarrow S_2$	325	0.1326	$\pi_{H-1} \rightarrow \pi^*_L$	52	
$S_0 \rightarrow T_7$	302	0.0000	$\pi_{H-3} \rightarrow \pi^*_L$	72	
$S_0 \rightarrow T_8$	287	0.0000	$\pi_{H-4} \rightarrow \pi^*_{L+3}$<br>$\pi_{H-1} \rightarrow \pi^*_L$	18<br>21	
$S_0 \rightarrow S_3$	287	0.1133	$\pi_{H-1} \rightarrow \pi^*_{L+3}$<br>$\pi_H \rightarrow \pi^*_{L+2}$	29<br>48	
$S_0 \rightarrow T_9$	285	0.0000	$\pi_{H-5} \rightarrow \pi^*_{L+2}$	28	
$S_0 \rightarrow S_4$	284	0.0187	$\pi_H \rightarrow \pi^*_{L+3}$	43	
$S_0 \rightarrow T_{10}$	276	0.0000	$\pi_{H-6} \rightarrow \pi^*_{L+1}$	15	
$S_0 \rightarrow S_5$	270	0.0120	$\pi_H \rightarrow \pi^*_{L+1}$	41	
$S_0 \rightarrow S_6$	262	0.3274	$\pi_{H-2} \rightarrow \pi^*_L$	45	
$S_0 \rightarrow S_7$	254	0.0011	$\pi_H \rightarrow \pi^*_{L+7}$	24	
$S_0 \rightarrow S_8$	248	0.1325	$\pi_{H-3} \rightarrow \pi^*_L$	80	
$S_0 \rightarrow S_9$	245	0.0014	$\pi_H \rightarrow \pi^*_{L+4}$	45	
$S_0 \rightarrow S_{10}$	241	0.0595	$\pi_H \rightarrow \pi^*_{L+5}$	38	
$S_1 \rightarrow S_0$	534	2.1162	$\pi_H \rightarrow \pi^*_L$	93	488

Table S18: Absorption and emission wavelengths (λ), oscillator strength (f) and molecular orbitals of the **elongated DMA-QT** conformer in Toluene (CPCM) calculated by the WB97XD/6-31+G(d)//B3LYP/6-31+G(d) model, together with the experimental absorption and emission maxima.

Transition	λ_{th}/nm	f	MOs	%	λ_{exp}/nm
$S_0 \to T_1$	860	0.0000	$\pi_H \to \pi^*_L$	81	
$S_0 \to T_2$	539	0.0000	$\pi_H \to \pi^*_{L+1}$	49	
$S_0 \to S_1$	429	2.3597	$\pi_H \to \pi^*_L$	88	436
$S_0 \to T_3$	389	0.0000	$\pi_{H-2} \to \pi^*_L$<br>$\pi_{H-1} \to \pi^*_{L+1}$	26<br>25	
$S_0 \to T_4$	342	0.0000	$\pi_{H-1} \to \pi^*_{L+2}$<br>$\pi_H \to \pi^*_{L+3}$	39<br>45	
$S_0 \to T_5$	342	0.0000	$\pi_{H-1} \to \pi^*_{L+3}$<br>$\pi_H \to \pi^*_{L+2}$	40<br>44	
$S_0 \to T_6$	333	0.0000	$\pi_{H-2} \to \pi^*_{L+1}$	25	
$S_0 \to S_2$	328	0.0409	$\pi_{H-1} \to \pi^*_L$<br>$\pi_H \to \pi^*_{L+1}$	41<br>50	
$S_0 \to T_7$	297	0.0000	$\pi_{H-3} \to \pi^*_L$	69	
$S_0 \to S_3$	287	0.0144	$\pi_{H-1} \to \pi^*_{L+3}$<br>$\pi_H \to \pi^*_{L+2}$	29<br>50	
$S_0 \to T_8$	286	0.0000	$\pi_{H-4} \to \pi^*_{L+2}$<br>$\pi_{H-4} \to \pi^*_{L+3}$	27<br>25	
$S_0 \to T_9$	285	0.0000	$\pi_{H-4} \to \pi^*_{L+3}$<br>$\pi_{H-4} \to \pi^*_{L+2}$	32<br>36	
$S_0 \to S_4$	284	0.0737	$\pi_{H-1} \to \pi^*_{L+2}$<br>$\pi_H \to \pi^*_{L+3}$	31<br>43	
$S_0 \to T_{10}$	278	0.0000	$\pi_{H-6} \to \pi^*_{L+1}$	17	
$S_0 \to S_5$	273	0.0105	$\pi_{H-1} \to \pi^*_L$<br>$\pi_H \to \pi^*_{L+1}$	35<br>33	
$S_0 \to S_6$	261	0.3137	$\pi_{H-2} \to \pi^*_L$	43	
$S_0 \to S_7$	259	0.0007	$\pi_H \to \pi^*_{L+7}$	58	
$S_0 \to S_8$	245	0.0000	$\pi_H \to \pi^*_{L+4}$	50	
$S_0 \to S_9$	243	0.1136	$\pi_{H-3} \to \pi^*_L$	84	
$S_0 \to S_{10}$	241	0.05618	$\pi_H \to \pi^*_{L+5}$	37	
$S_1 \to S_0$	525	2.3241	$\pi_H \to \pi^*_L$	93	488

3. *Femtosecond transient absorption and fluorescence up conversion*

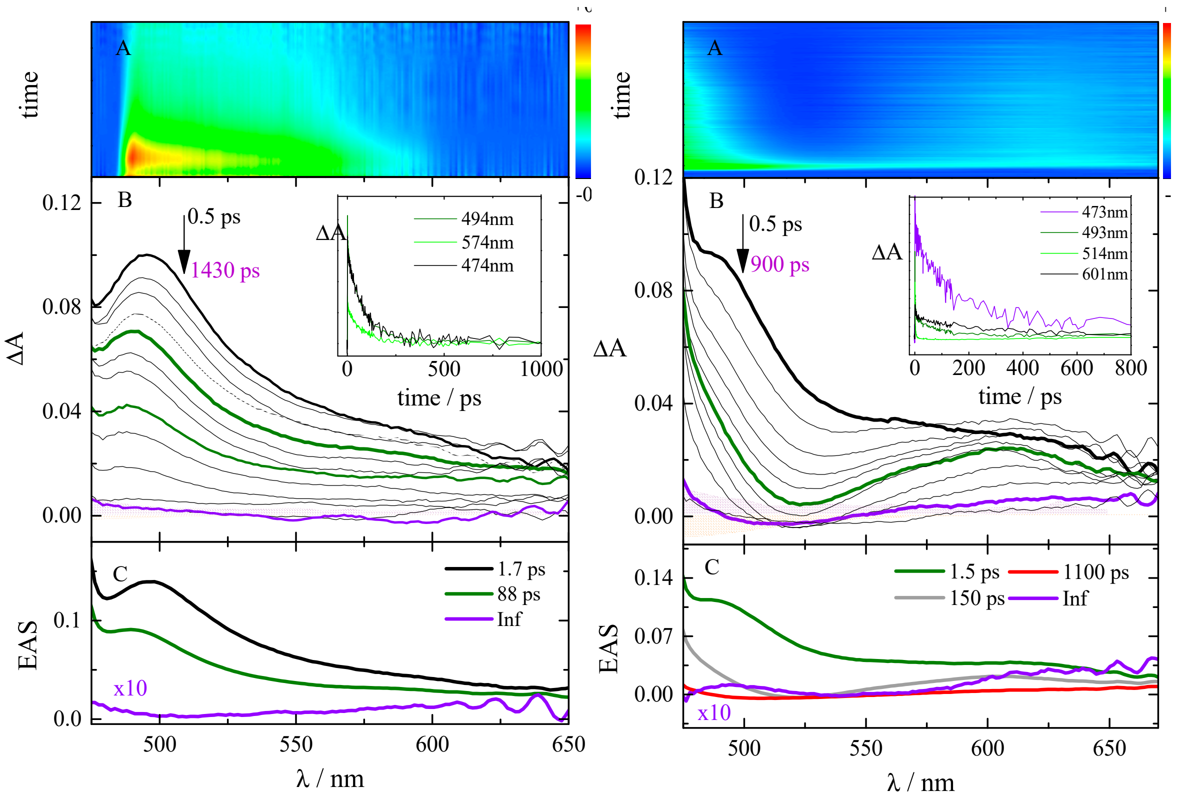

Figure S6: Femtosecond transient absorption measurements of compound **DMA-QP** in Tol (left) and DMF (right) obtained by pump-probe experiment (λ_{exc}= 400 nm).

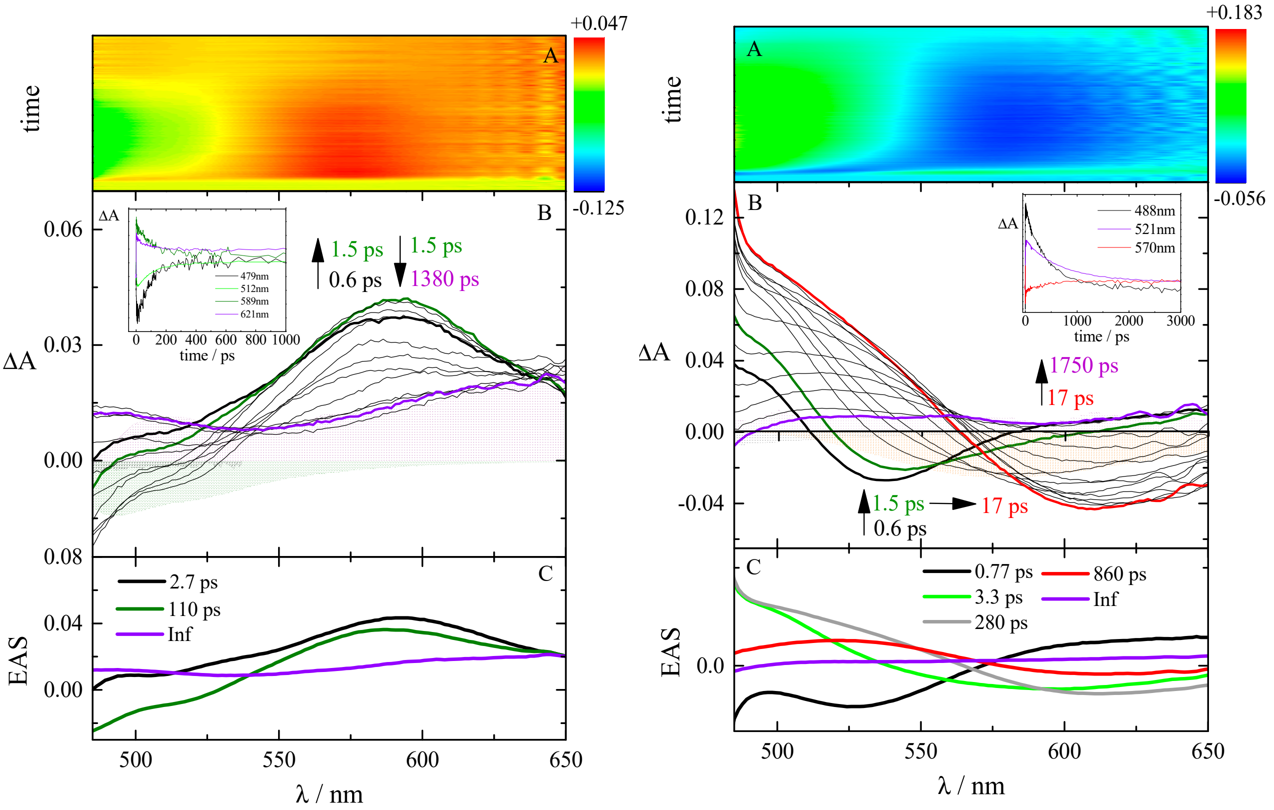

Figure S7: Femtosecond transient absorption measurements of compound **DMA-QF** in Tol (left) and DMF (right) obtained by pump-probe experiment (λ_{exc}= 400 nm).

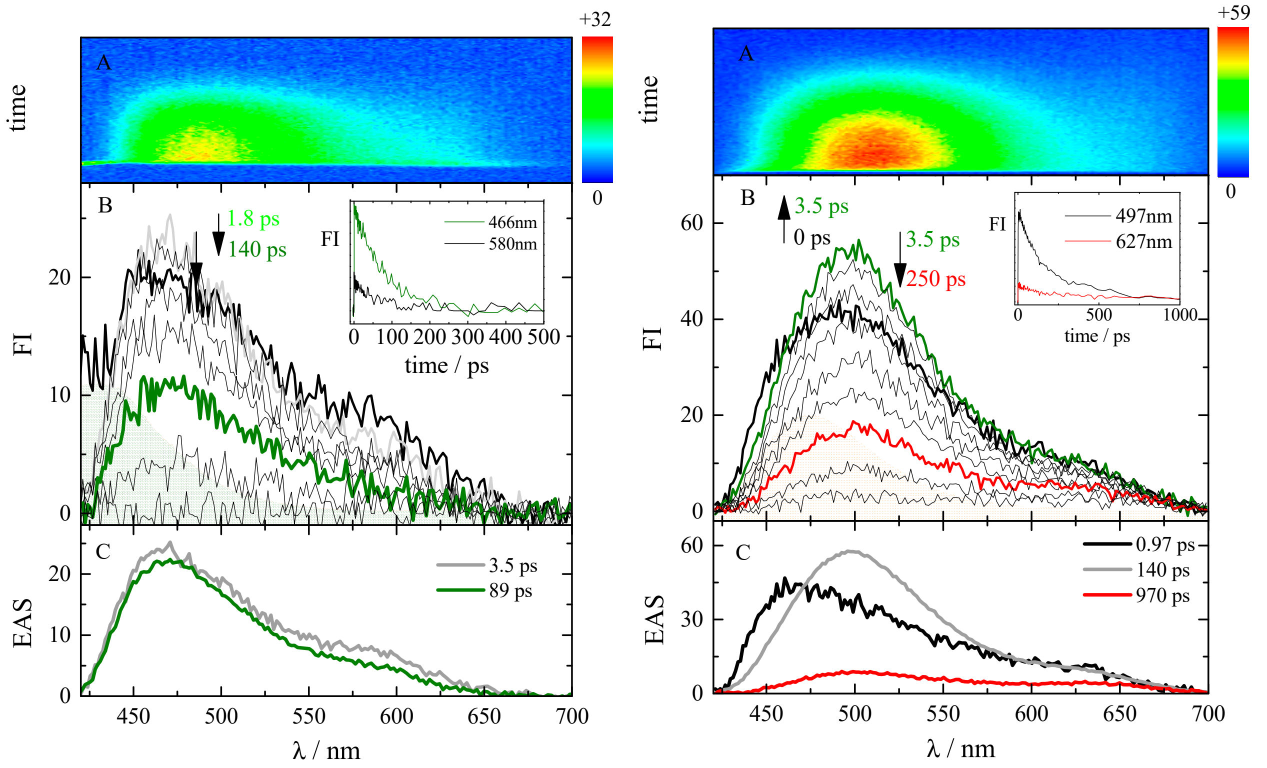

Figure S8: Femtosecond Up conversion measurements (λ_{exc}= 400 nm) of compound **DMA-QP** in Tol (left) and DMF (right).

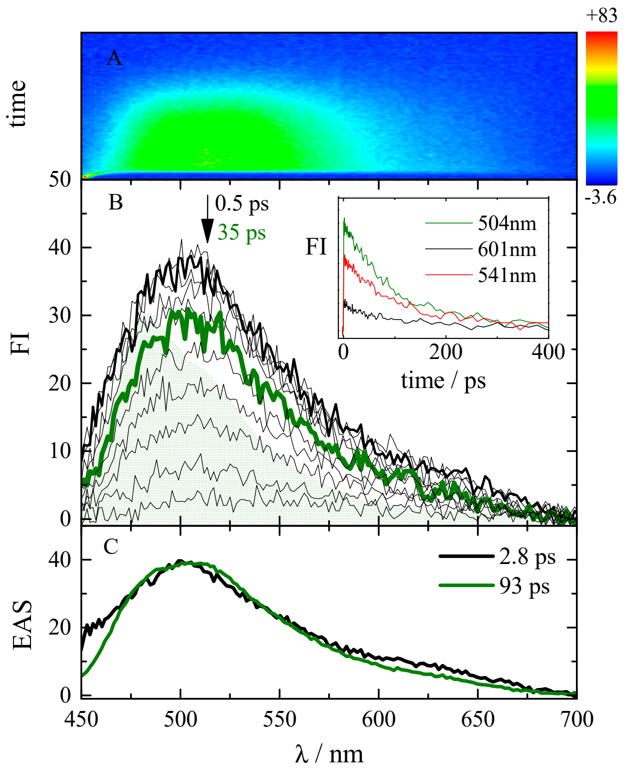

Figure S9: Femtosecond Up conversion measurements (λ_{exc}= 400 nm) of compound **DMA-QF** in Tol.

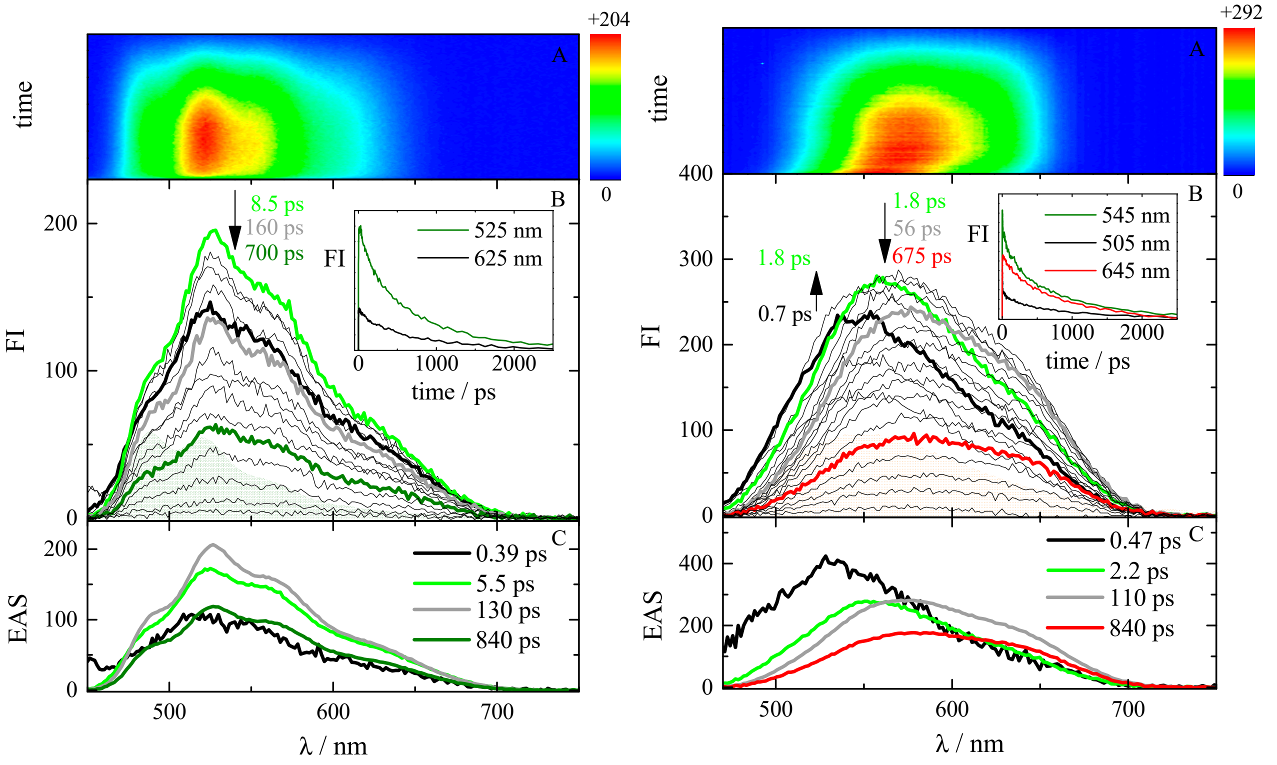

Figure S10: Femtosecond Up conversion measurements (λ_{exc}= 400 nm) of compound **DMA-QT** in Tol (left) and DMF (right).

Table S19: Fs-TA and FUC results of Global Analysis of compounds **DMA-QP**, **DAQF** and **DMA-QT** in Tol and DMF obtained by exciting at 400 nm.

Compound	Solvent	TA		FUC		Assignment
		λ (nm)	τ (ps)	λ (nm)	τ (ps)	
	Tol	495(+)	1.7	470	3.5	Solv.
		490(+), 575(+)	**88**	470, 595^{sh}	**89**	$\mathbf{S_1}$
		<480(+)	**Inf**			$\mathbf{T_1}$
**DMA-QP**	DMF	490(+)	**1.5**	470	**0.97**	**Solv.$_{i.}$, $S_{1, LE}$**
		<480, 600(+)	150	500	140	SR
		<480, 520(-), 600(+)	**1100**	500, 650^{sh}	**970**	$\mathbf{S_{1, ICT}}$
		<480, 650(+)	**Inf**			$\mathbf{T_1}$
	Tol	<490(+), 580(+)	2.7	500	2.8	Solv.
		520 (-), 570(+)	**110**	505	**93**	$\mathbf{S_1}$
		<480(+), >650(+)	**Inf**			$\mathbf{T_1}$
**DMA-QF**	DMF	<480, 525(-)	**0.77**	515	**0.68**	**Solv.$_{i.}$, $S_{1, LE}$**
		<480, 580(-)	3.3	570	3.0	Solv.$_{d.}$
		<480, 610(-)	280	590	270	SR
		<480, 620(-)	**860**	600	**830**	$\mathbf{S_{1, ICT}}$
		<480, >650(+)	**Inf**			$\mathbf{T_1}$
	Tol	520(-), 700(+)	0.90	513	0.39	Solv.$_{i.}$
		525(-), 700(+)	6.4	525	5.5	Solv.$_{d.}$
		525(-), 700(+)	130	525	130	SR
		525(-), 700(+)	**900**	525, 575^{sh}	**840**	$\mathbf{S_1}$
**DMA-QT**		610(+)	**Inf**			$\mathbf{T_1}$
	DMF	535(-), 665(+)	**0.57**	535	**0.47**	**Solv.$_{i.}$, $S_{1, LE}$**
		545(-), 700(+)	3.1	555	2.2	Solv.$_{d.}$
		550(-), 750(+)	170	575, 635^{sh}	110	SR
		555(-), >750(+)	**1000**	595	**840**	$\mathbf{S_{1, ICT}}$
		610(+)	**Inf**			$\mathbf{T_1}$

4. Nanosecond transient absorption

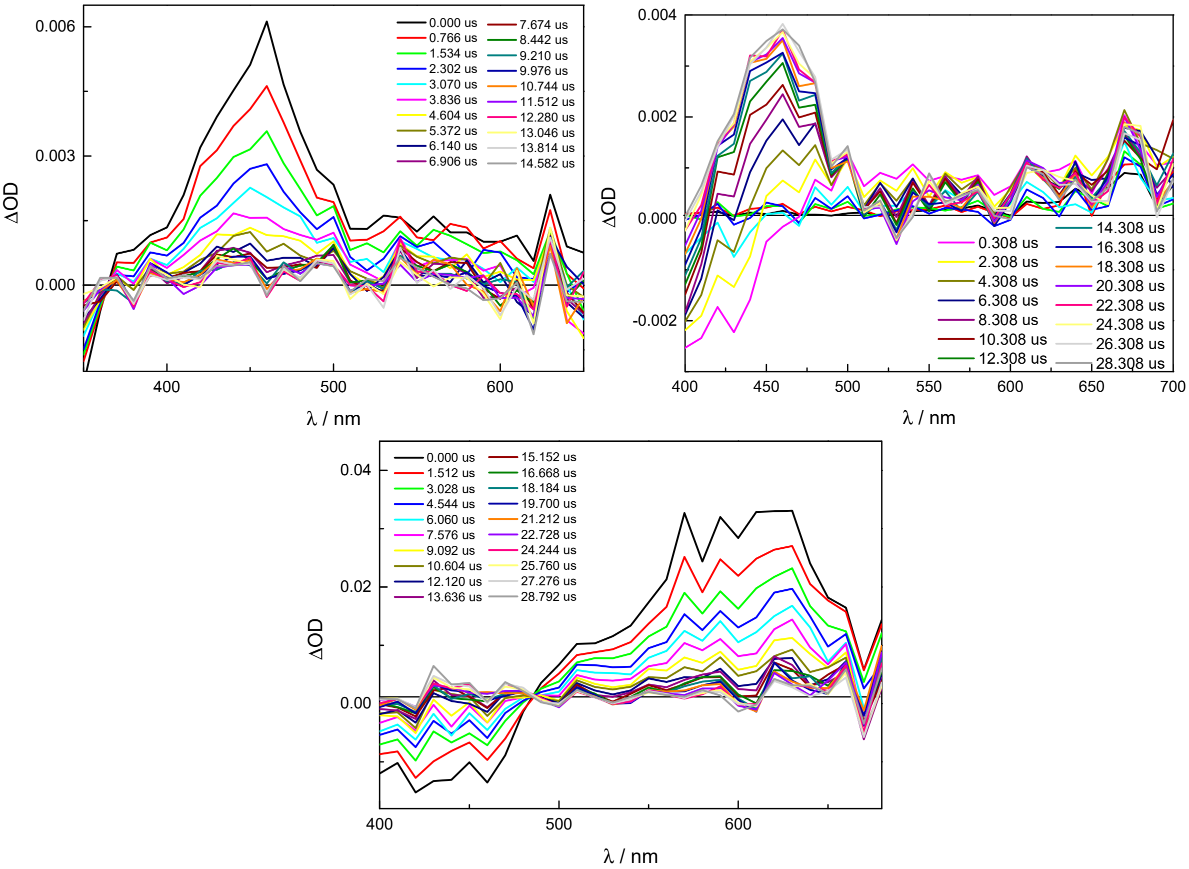

Figure S11: Nanosecond transient absorption spectra of **DMA-QP** (top-left), **DMA-QF** (top-right), and **DMA-QT** (bottom) in de-aerated DMF obtained by laser flash photolysis (λ_{exc} = 355 nm).

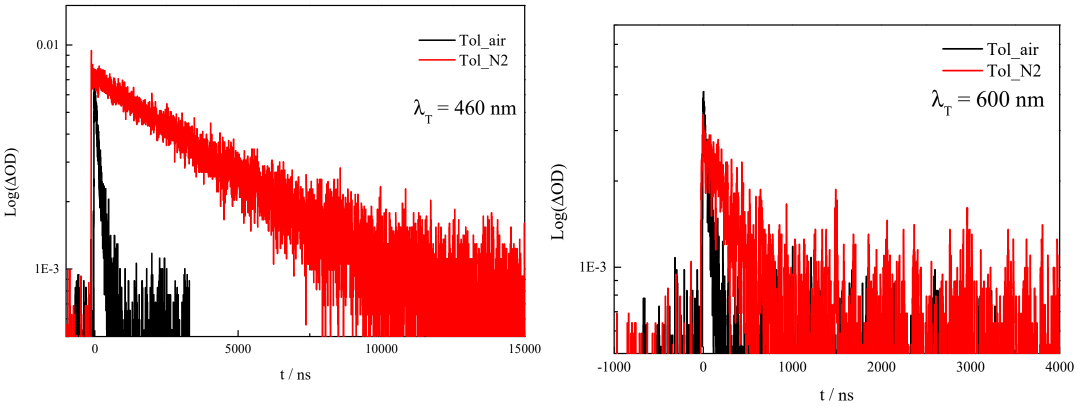

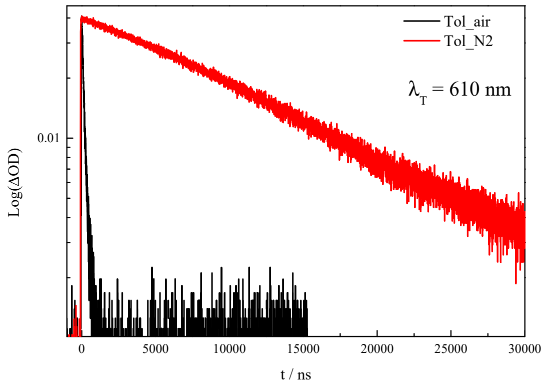

Figure S12: Comparison between triplet lifetimes in aerated and de-aerated Tol recorded at representative λ_T for **DMA-QP** (top-left), **DMA-QF** (top-right), and **DMA-QT** (bottom) obtained by laser flash photolysis (λ_{exc} = 355 nm).

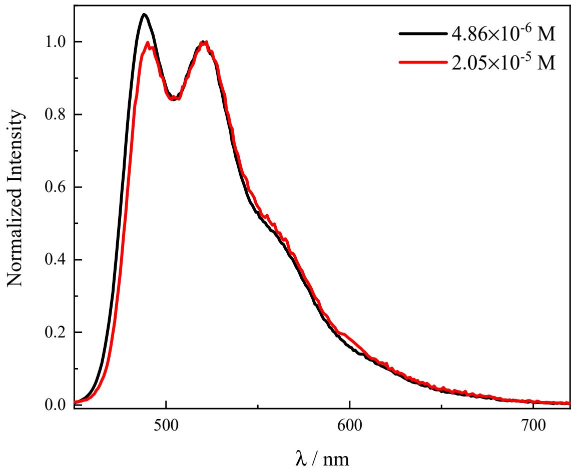

Figure S13: Concentration effect on the emission spectra of **DMA-QT** in Tol.

5. Singlet oxygen phosphorescence

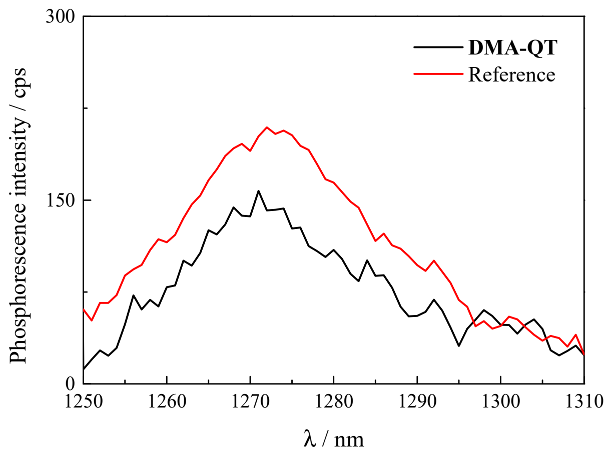

Figure S14: Singlet oxygen phosphorescence spectra of **DMA-QT** in Tol and of the reference (Phenalenone in Tol) by exciting each compound at the relative absorption maximum.

6. ^{1}H and ^{13}C NMR spectra ($CDCl_3$) of new compounds DMA-QT and DMA-QF

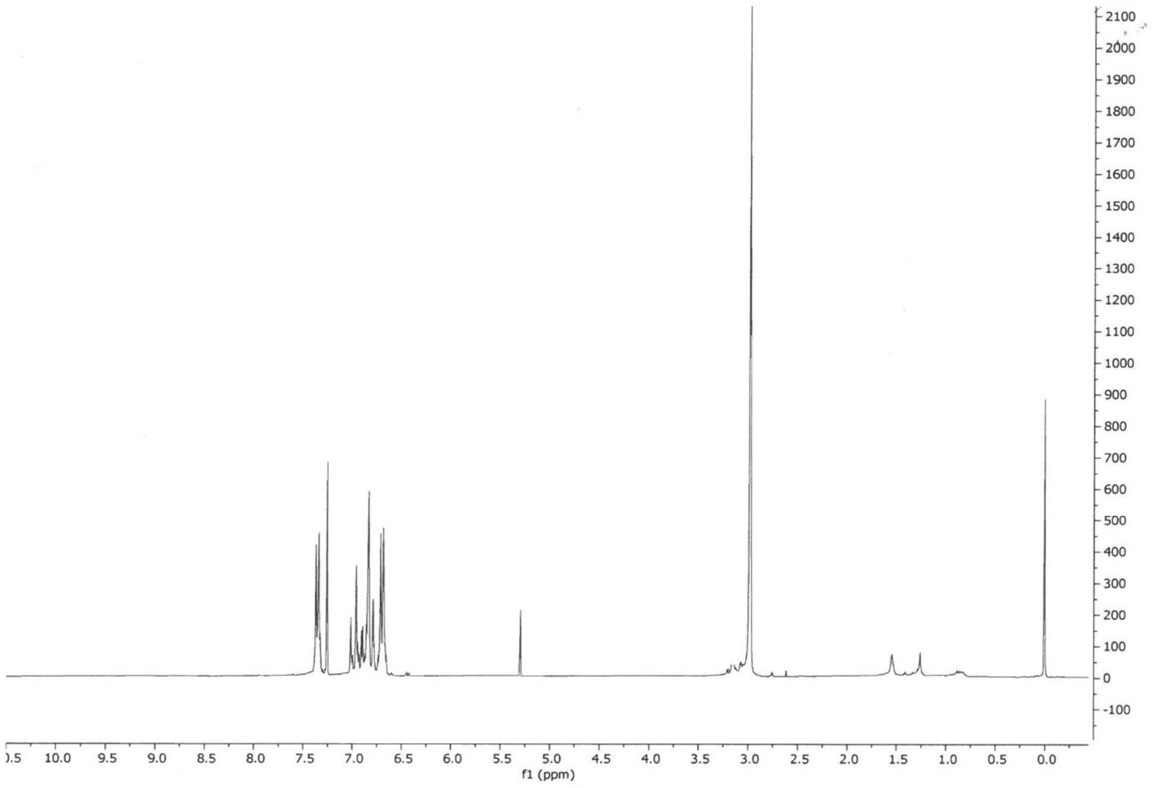

FigureS15. ^{1}H NMR spectrum ($CDCl_3$) of **DMA-QT**.

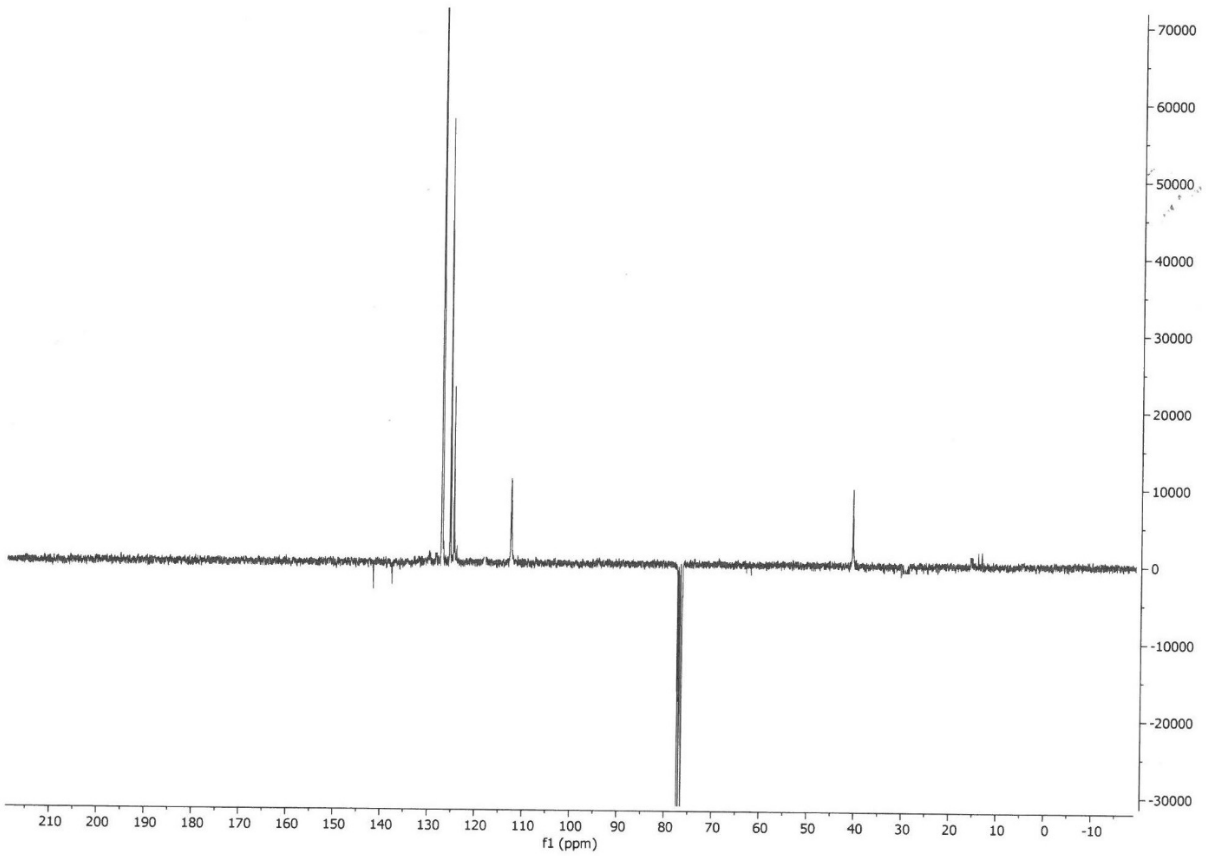

FigureS16. ^{13}C NMR spectrum ($CDCl_3$) of **DMA-QT**.

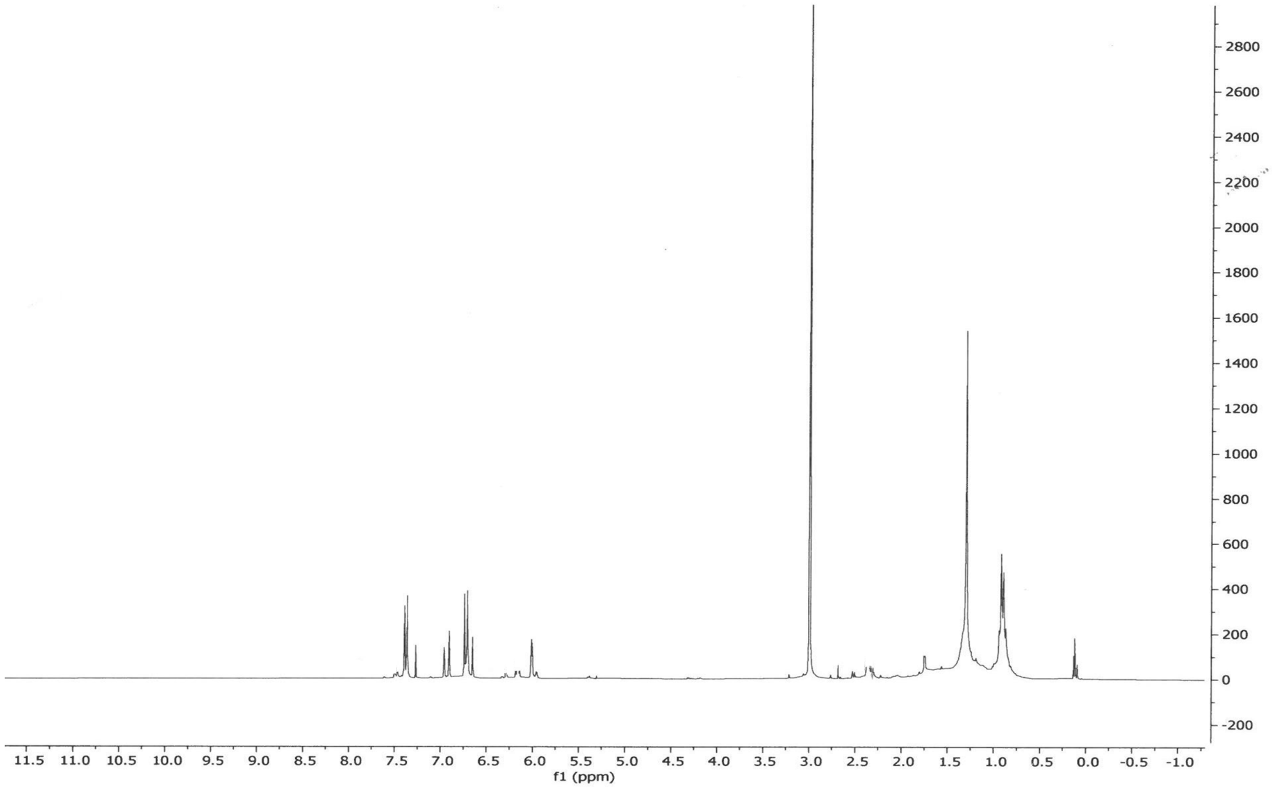

FigureS17. ^{1}H NMR spectrum ($CDCl_3$) of **DMA-QF**.

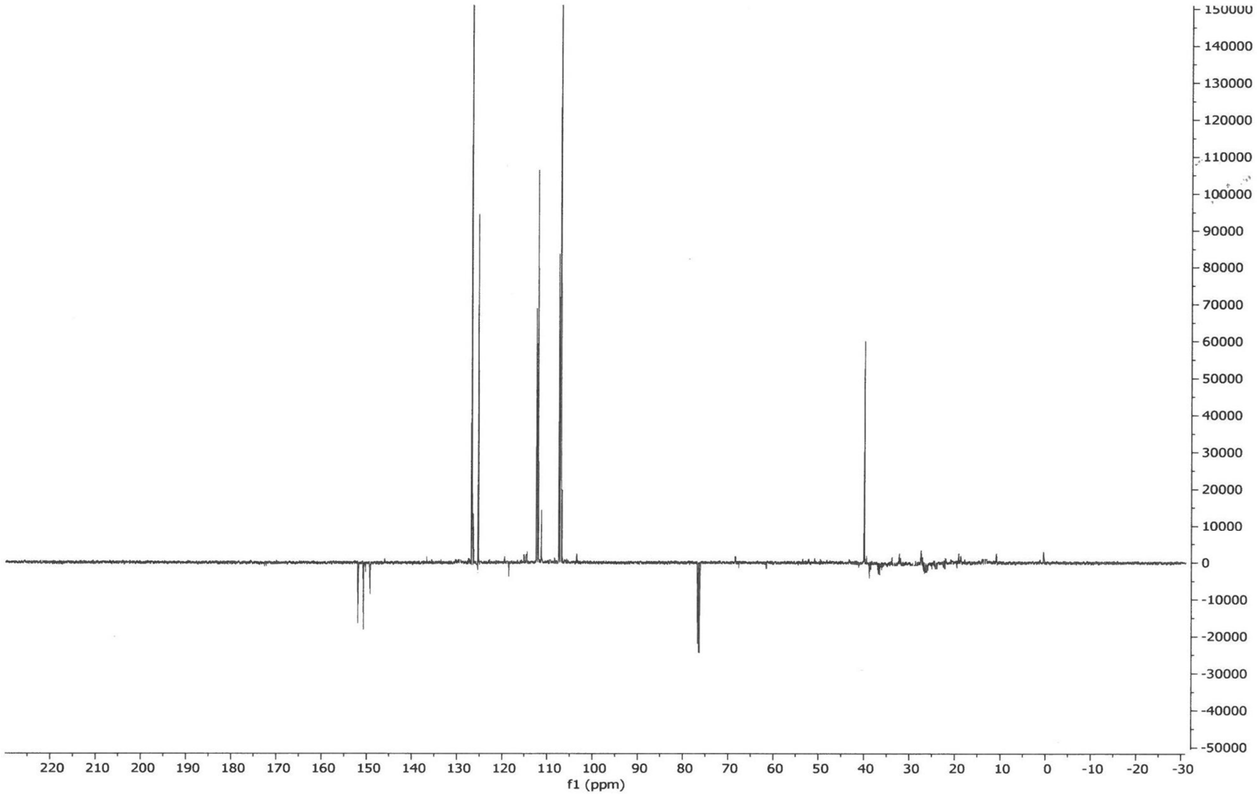

FigureS18. ^{13}C NMR spectrum ($CDCl_3$) of **DMA-QF**.
